# Attitudes of Patients with Non-Psychotic Mental Disorders Towards Cannabis After Its Legalization—Comparison with Patients Before Legalization

**DOI:** 10.3390/brainsci16070730

**Published:** 2026-07-11

**Authors:** Michael Specka, Josef Rabl, Udo Bonnet, Marah Rosner, Anna Schmitt, Norbert Scherbaum

**Affiliations:** 1 LVR-University Hospital Essen, Department of Psychiatry and Psychotherapy, Faculty of Medicine, University of Duisburg-Essen, 45147 Essen, Germany; michael.specka@uni-due.de (M.S.); udo.bonnet@uni-due.de (U.B.); marah.rosner@hhu.de (M.R.);; 2 Evangelical Hospital Group Augusta Ruhr, Department of Mental Health, Evangelical Hospital Castrop-Rauxel, Academic Teaching Hospital of University of Duisburg-Essen, 44577 Castrop-Rauxel, Germany

**Keywords:** mental health patients, recreational cannabis, cannabis legalization, attitudes towards cannabis

## Abstract

**Background:** The illegal status of recreational cannabis (RC) is often considered a barrier to its use. RC was partially legalized in Germany in 2024. This study investigated whether attitudes towards cannabis were different in patients surveyed after recreational cannabis legalization (RCL), compared with patients surveyed before RCL. **Method:** In 2022 (pre-RCL) and 2024/2025 (post-RCL), patients without psychosis and without substance use disorders from in-patient and day clinic wards of a psychiatric university hospital were interviewed using a standardized questionnaire. **Results:** Included were *n* = 143 patients pre-RCL and *n* = 117 post-RCL, with 84% presenting with affective disorders. Only 11.2% in the pre-RCL group, and 1.7% in the post-RCL group indicated the illegal status of RC as the main reason for cannabis abstinence. Attitudes towards cannabis with regard to health concerns, social distance, general rejection of (illicit) drugs, effects of drug education, or observation of negative consequences in others were not statistically significantly different between groups. There was a marked and statistically significant reduction in fear of being investigated by the police because of RC consumption. The suggestion to buy cannabis for personal use in specialized shops was completely rejected by 58.7% of the pre-RCL and by 71.8% of the post-RCL group. **Conclusions:** Compared with pre-RCL data, concerns about negative legal consequences of RC use were lower during the first year post-RCL, but readiness to legally obtain recreational cannabis was not increased. There were no indications that other attitudes about cannabis were affected in the short term.

## 1. Introduction

In recent years, several countries worldwide have introduced recreational cannabis legalization (RCL). The impact of RCL on the prevalence of cannabis use, on public health, and on mental health outcomes has been a subject of debate among researchers and policymakers. Arguments against the legalization of cannabis include its risks for individual health, such as acute and chronic cognitive impairment [1], negative effects on adolescent brain development, and associations with psychiatric disorders such as drug induced psychosis, schizophrenia and cannabis dependence [2,3,4]. Regarding the prevalence of cannabis use, some studies found no association between RCL and changes in cannabis use among youth in European countries, Uruguay, the USA or Canada, while other studies have reported increases in cannabis use among youth and adults [5]. Positive correlations between cannabis availability and consumption appeared most pronounced among those groups who have been less exposed to cannabis before legalization [6].

One subpopulation for whom RCL could be of particular concern are individuals with mental health problems. Cross-sectional studies have consistently shown increased rates for nonmedical (recreational) cannabis use and cannabis use disorders in people with mental health problems, compared with the general population [7,8,9,10,11]. In people with mental health problems, regular cannabis use can lead to the worsening of depression, anxiety and psychotic symptoms [12,13,14]; in patients with depression, cannabis use may impede symptom improvement, and it is associated with poorer recovery [15,16]. 

In Germany, the production, possession and trade of cannabis, but not its use, was prohibited by law since 1929. However, since 1994, following a ruling by the Federal Constitutional Court, prosecutors could routinely refrain from prosecuting the possession of small amounts of cannabis for personal use. In 2017, in Germany the prescription of medical cannabis was permitted for patients with severe diseases who had previously unsuccessfully been treated with state-of-the-art treatment. The political parties which formed a new government in 2021 agreed upon decriminalization of recreational cannabis. Cannabis possession for personal use should become legal, as well as self-cultivation at home or as a member of a highly regulated “cannabis cultivation club”. Authorization of specialized, licensed cannabis shops was announced, but in the final legislation it was only included as a future second possible step. RCL came into effect on 1 April 2024. During the discussion about RCL, medical experts and medical societies in Germany expressed concerns about the risk that the legalization of cannabis would lead to higher availability of cannabis which in turn would lead to a higher prevalence of cannabis use with its negative effects on mental health, especially for youth and adolescents. In addition, it was argued that legalization would decrease the risk perception by consumers and the public [17].

One major assumption within this discussion therefore was that the illegal status of cannabis played a major role in preventing individuals from its recreational use. The validity of this assumption is particularly important with respect to people with mental health problem, who have an increased risk of negative effects of cannabis use. 

However, little is known about the perspectives of mental health patients on RCL, particularly of those without psychotic disorders. Nevertheless, it is plausible that health-related considerations are already salient for this population—independent from the legal status of cannabis. Another well-known factor in initiating drug use or maintaining abstinence is peer influence, with friends and social networks playing an important role [18]. In addition, individuals may choose not to use cannabis because of social or moral norms, such as a general desire to avoid psychotropic drug use or to keep away from stigmatised ‘drug scenes’; these rules and norms may have been transmitted by significant others, such as family members, or through public health education, respectively.

Using data from two cross-sectional surveys carried out with psychiatric patients before and after change in cannabis legislation in Germany, the present study explores the attitudes of psychiatric in-patients without organic psychosis or schizophrenia towards cannabis use, and their readiness to legally obtain cannabis. We compared these data with those from a group of patients examined before legalization. Additionally, factors are assessed which presumably prevent patients from the use of cannabis.

## 2. Materials and Methods

### 2.1. Setting

The study was carried out in the psychiatric hospital of a university hospital in the city of Essen, Germany. The city with its population of approximately 600,000 inhabitants is located in the center of the large urban Ruhr area (5.2 million inhabitants). 

### 2.2. Inclusion Criteria

During the two study periods (June to December 2022 and October 2024 to April 2025, respectively), patients (aged between 18–60 years) were recruited from the study hospital’s in-patient and day clinic wards. Patients with a history of cannabis dependence in the past 12 months were excluded. Further exclusion criteria were substance-related disorders (except nicotine), psychotic disorders, and dementia. In addition; patients were excluded if their current psychiatric symptoms (e.g., severe depressive symptoms) prevented participation; and if their German language capabilities were not sufficient. Severity of psychiatric symptoms was not assessed formally; instead, the staff on the ward were asked whether patients were eligible for participation in their opinion. In addition, patients were explicitly asked whether they felt well enough to participate, and they were informed that they could terminate the interview at any time without negative consequences.

### 2.3. Measurements

Data were collected using a self-designed questionnaire which comprised socio-demographic characteristics; history of cannabis use; history of use of other illicit drugs; history of misuse of prescribed medication (i.e., intake of higher doses than prescribed, and/or purchase and use without prescription; explicitly mentioned here were: Gabapentinoids, opiate analgesics, benzodiazepines/Z-drugs, and medication for attention deficit/hyperactivity disorder). Furthermore, the questionnaire contained nine statements about expected negative consequences of own cannabis use and 13 statements about experiences with and attitudes towards cannabis. The items were developed using clinical expertise, existing literature on drug use, and the ongoing public debate surrounding RCL. It was considered important that patients could rate the personal significance of known risks associated with cannabis use (e.g., concerning mental and physical health), as well as social and environmental factors that influence cannabis use (e.g., drug education, legislation, social norms, availability, or social proximity to cannabis users). Degree of agreement was indicated on a 7-point scale (from “not at all true” to “completely true”). For all items, higher scores meant a more negative attitude or belief, or greater distance towards cannabis. For the remainder of this article, we will refer to these items using the term “attitudes”.

In addition, participants were asked for the most important personal reason for not using cannabis. The answer to this was given in free format.

Finally, patients from both groups were informed that in the future cannabis might be purchased in specialized licensed shops, as was publicly discussed at the time of the first data collection. They were asked whether they agreed with the launch of such shops (agree/do not agree/undecided), and whether they would buy cannabis there (no, perhaps, probably, certainly). Patients in 2024/2025 also received a short description of the new legislation, which permitted growth of cannabis plants for personal use, or in the context of a cannabis cultivation club. For both sources of supply, they should indicate whether they would use it in the future (no, perhaps, probably, certainly). During a pilot phase, the questionnaire was distributed to patients from the target group to check for clarity, comprehensiveness and missing information. Based on patient feedback, a revised version was created which was used in the main study. No further formal validation of the questionnaire was performed. The original questionnaire and an English translation can be found in Supplementarys S1 and S2.

### 2.4. Procedure

Data were collected by doctoral medical students. Patients were informed about the study background, the data collection process, and data protection measures, before they gave written informed consent. The doctoral students instructed the participants about how to use the questionnaire, and answered additional questions.

### 2.5. Data Analysis

Reasons for not using cannabis were clustered thematically by one author (MS) and a specialist in psychiatry who was experienced in the treatment of mental disorders, including substance use disorders. The categorization procedure was bottom-up and loosely based on the metaplan technique for visualizing and organizing information [19]. Collected statements were presented on separate sheets. A first statement was presented, then a subsequent statement. It was decided whether the second statement had a topic similar to the first one. If yes, both statements were clustered. If not the second statement was dealt with as belonging to a new category. For each subsequent statement, it was decided whether it could be put in an existing cluster or not. Statements for which both raters could not reach immediate agreement were temporarily separated. After all statements had been processed, the clusters received headings and it was decided whether any clusters could be merged and whether separate statements could be added to a cluster. We expected 3–8 themes (categories) to finally emerge [20]. Since the procedure was consensus-based, no measurements of interrater agreement could be calculated. 

Statistical tests for mean differences were Welch-t-tests for independent groups. Group comparisons with ordinal-scaled variables were made using Mann–Whitney U-test; for categorical variables, using Chi^2^ tests. The threshold for a statistically significant result was 0.05. Degree of association is expressed by odds ratios, Somer’s D (which expresses the associations of cohort with ordinal scaled variables and can have values between +1 and −1 [21]), and mean differences, respectively, with 95% confidence intervals.

Analyses were made with IBM SPSS statistics 31 [22].

## 3. Results

### 3.1. Sample

In 2022, *n* = 187 patients aged 18 to 60 and without a substance dependence diagnosis at entry were screened for eligibility. *n* = 5 of these could not be included because of the severity of their psychiatric symptoms, *n* = 1 because of language problems, *n* = 29 patients refused participation, and *n* = 9 patients who had agreed in participation did not return the questionnaire. Thus *n* = 143 patients were included.

In 2024/2025, *n* = 222 patients were screened. Of these, *n* = 68 refused participation, *n* = 33 did not return their questionnaire, and *n* = 4 (1.7%) received a substance dependence diagnosis at discharge. Thus, *n* = 117 patients were included. According to the medical student who collected the data, the markedly higher refusal rate in 2024/2025 was due to another study conducted concurrently on the same wards. For many patients, participating in two projects felt like too much effort.

Sociodemographic characteristics, diagnoses and drug use history appeared very similar between groups (Table 1 and Table 2). The majority were female; age was between 18 and 60 years with a mean of 35 years. In the majority of cases an affective disorder was the main diagnosis (85.3% 82.9%, respectively). More than 60% of the patients in both groups had used cannabis at least once during lifetime, though very frequent use (more than 50 times during lifetime) was reported by a minority (16.1% vs. 11.1%, respectively). About a quarter of patients in both groups had used cannabis during the previous 12 months.

### 3.2. Attitude Questionnaire

Responses of both cohorts to the 22 items concerning attitudes and beliefs with regard to cannabis are displayed in Table 3. Items are grouped according to the topics which were considered important when constructing the questionnaire. 

Both groups showed the relatively highest agreement with statements concerning negative effects of cannabis on mental health and on cognitive performance; with rejection of contact with “drug scenes”; and with general rejection of drugs. Possible trouble at work and fear of losing the driver’s license were also pronounced in both groups, while fear of being investigated by the police was much higher in 2022 than in 2024/2025 (mean 4,5 versus 3.6 points). This was also the only statistically significant group difference (*p* < 0.001), except “As a non-smoker, I avoid smoking cannabis cigarettes (joints)”, which also showed a significantly (*p* = 0.028) lower mean in 2024/2025, compared with 2022. 

In order to account for possible confounding in this observational design, comparisons were repeated with age, gender, main diagnosis, current tobacco use, lifetime cannabis exposure, recent cannabis use, employment status, education and migrant background as covariates in multiple regression analyses. Results were similar to those from the Welch tests, i.e., group (2022 vs. 2024/2025) showed a statistically significant regression coefficient (*B* = −1.06, *s.e.* = 0.29, *p* < 0.01) for fear of being investigated by the police, and the remaining analyses indicated no statistically significant group effect (see Supplementary S3). Regarding “reject cannabis (joints) as a non-smoker”, the group effect was no longer statistically significant (*B* = 0.57, *s.e.* = 0.40, *p* = 0.16).

### 3.3. Attitudes to Legal Procurement of Cannabis

Large proportions of both samples endorsed the idea of legal purchase of cannabis in licensed shops, or at least did not clearly reject this (Table 4). The difference between groups was not statistically significant (*p* = 0.71, Mann–Whitney U-test). Nevertheless, the majority in both samples would not consider buying cannabis in such shops. The rejection of this was markedly more frequent (71.8%) in the 2024/2025 cohort than in the 2022 cohort (58.7%). This difference was statistically significant (*p* = 0.02, U-test). 

In addition, very few patients in the 2024/2025 group expressed readiness to obtain cannabis as permitted by the new legislation, i.e., by home growing (0.9%) or through membership in a cannabis social club (2.6%).

### 3.4. Most Important Reasons for Not Using Cannabis

**Figure 1:**
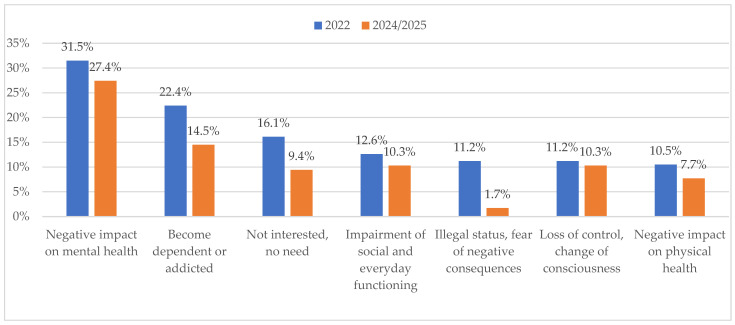
Main reasons for not using cannabis (categorized free answers). Reasons are displayed if reported by at least 10% of patients in one of the cohorts.

Patients were asked for a main reason for not using cannabis. Most frequent were concerns about the negative impact on mental health (e.g., “worsening of my mental health condition”, “afraid of mental damage”, “afraid of psychosis”), and fear of becoming addicted (Figure 1). Relative frequencies of both topics showed no significant group effect (*p* = 0.47 for mental health concerns, *p* = 0.11 for fear of addiction; Chi^2^ test). Also, the other types of answers revealed no statistically significant group effect (all *p* > 0.1), with the exception of “illegal status of cannabis, fear of negative consequences” (e.g., “illegality”, “being investigated by the police”, “legal consequences”), which had been mentioned by 1 in 8 patients in 2022, and even fewer (*n* = 2) patients in 2024/2025 (*p* = 0.003). Odds ratios (95% c.i.) for comparisons between groups were: Negative impact on mental health, 0.82 [0.48; 1.41]; Become dependent or addicted: 0.59 [0.31; 1.13]; Not interested, no need, 0.54 [0.26; 1.16]; Illegal status, fear of negative consequences, 0.14 [0.03; 0.61]; Negative impact on physical health, 0.71 [0.30; 1.69]; Impact on social and everyday functioning 0.79, [0.37; 1.72]; Loss of control, change in consciousness, 0.91 [0.41; 2.00].

### 3.5. Association of Cannabis Related Attitudes and of Reasons for Abstinence with Recent Cannabis Use

About 75% of patients in each sample indicated complete abstinence from cannabis within the previous 12 months. Two patients (from the 2024/2025 cohort) had not answered this question and are not included in the subsequent analyses. 

Cannabis non-users expressed more concerns than recent users on nearly every item (Supplementary S4). In particular, items describing social distance towards cannabis and cannabis users, and items expressing general rejection of (illicit) drugs showed mean differences of 1.5 points or more on the 1–7-point scale. With regard to health concerns, groups were much more similar, but nonusers still showed the stronger concerns, on average, and differences were statistically significant. The same was true for educational influences. With regard to fear of being investigated by the police, users and non-users differed by 0.6 points, which was not statistically significant. 

Regarding the most frequent reasons for not using cannabis, negative impact on mental health was stated most often both by last year’s abstainers as well as non-abstainers, and the difference in proportion was not statistically significant (Table 5). Fear of becoming addicted was the next important reason for abstainers and was stated by nearly one in four members in this group, while this reason was stated by only very few of last year’s cannabis users. The illegal status was mentioned only by a small fraction in both groups. (6.8% and 11.1%, respectively).

Finally, in both cohorts, strong rejection of purchase in cannabis shops was more frequent in patients who had not consumed cannabis within the last 12 months, compared with consumers (68.9% versus 30.2% in the 2022 cohort, and 80.0% versus 44.8% in the 2024/2025 cohort).

## 4. Discussion

The present study investigated the attitudes of two groups of psychiatric patients (both treated as inpatients or in day treatment)—one before RCL came into effect, one shortly afterwards—regarding their risk assessment of cannabis use and their personal willingness to use cannabis. Most patients were treated for affective disorders. Patients with organic psychosis or those with schizophrenia, schizotypal, or delusional disorders were not included, as were patients with a current diagnosis of abuse or dependence on cannabis or other drugs except nicotine. Socio-demographic profiles and drug use histories were very similar between the two patient groups: Typically, patients were of young or of middle age, and the majority were female. About one third were not employed, and nearly half of the sample were living alone. The proportion of smokers (43.3% in the pre-RCL and 38.5%, post-RCL sample) was considerably higher than in the general adult population (about 26% in males, and 20% in females [23]). This is a common observation in populations with mental health problems, and a major contributing factor for their reduced life expectancy [24].

The majority in both cohorts had used recreational cannabis during lifetime at least once, and a significant minority reported extensive experience with cannabis (here defined as 50 consumption episodes or more). In addition, 29% (pre legalization) and 23% (post-legalization) had used cannabis at least once during the preceding year.

The questionnaire was designed to assess the significance of legal status, perceived availability, social acceptability, perceived risk, and also personal willingness to use cannabis. These factors played also a role if patients were given the opportunity to indicate spontaneously reasons for not using cannabis. Already before RCL, legal status (fear of legal consequences) was mentioned by only 11% as reason for abstinence. In contrast, fear of a negative impact on mental health (about 30%) or of becoming addicted (20%) was by far more important.

The average member in both cohorts expressed a marked social distance to cannabis and to cannabis users. Mean scores did not differ statistically significantly between groups. This might indicate that, independently of its achieved legal status, cannabis was still negatively assessed as a “Droge” (a term reserved for potentially harmful psychotropic substances in the German language, without the second meaning of a medical drug). It remains to be seen whether this attitude changes with time, if the public becomes more accustomed to the legal availability of cannabis, and social contacts with cannabis users become more common. 

The mean scores for concerns about mental and physical health risks did also not differ statistically significantly between groups. Additionally, no significant group difference was observed for the (comparatively lower) mean scores for reservations towards cannabis because of education on cannabis and parental influence. In contrast, the item describing negative legal consequences of cannabis use showed a statistically significant lower level of agreement after RCL, compared with 2022. In this context, it is worth noting that in the federal state of North Rhine Westphalia, before 2024 possession of small amounts of cannabis (up to 10 g) led to a criminal complaint filed by the police, however for more than two decades, these offenses had no longer been further investigated [25]. Therefore, even if patients acknowledged the possibility of negative legal consequences from cannabis use pre-RCL, they might have assumed only low practical impact. 

To our knowledge, short-term differences in attitudes towards cannabis between pre- and post-RCL have not yet been studied in individuals with mental health disorders. Studies with samples not defined by their mental health status, all carried out in North America, have produced mixed evidence. Reports showed a decrease in perceived harm risks [26], a more positive general attitude towards cannabis [27], more-positive health perceptions of cannabis use [28], and a shift in perceived cannabis effects on mental health from slightly harmful to slightly beneficial. In other studies, though, no changes for “low perceived harm” [29], or even increases in arguments contrary to cannabis use [30], of perceived harmfulness [31], and of perceived risks for mental and cognitive health [32] were observed. A general population study from Canada reported increased social acceptability of recreational cannabis use and for trying cannabis, but also of perception of cannabis as risk for mental health and as potentially addictive. Non-users before legalization showed larger increases towards greater social acceptability of occasional and regular use, and a smaller increase in the perception of cannabis-related risks [33]. Thus, a complex pattern of results has emerged for the general population, with as yet no clear tendency towards trivializing possible harms. Our own study suggests that shortly after RCL, mental health patients without psychosis or addiction problems held attitudes towards cannabis which were very similar to those investigated before RCL. Furthermore, nearly all areas of concern (e.g., health, social distance, general rejection of drugs) were seemingly not different between groups. Beyond possible attitude changes, evidence from the USA and Canada suggests a positive association of recreational cannabis legalization with increased commercial supply and increased prevalence of cannabis use in adults. Evidence on associations with psychiatric disorders has been mixed, but with regard to psychosis, there could be an increase in new cases and of hospitalizations. In contrast, little evidence exists that legalization in Europe and other parts of the world has been associated with more cannabis use or more psychiatric disorders [34].

In sum, evidence regarding the effects on RCL varies widely, partly due to variations in the populations studied (e.g., adolescents versus adults, individuals with mental health problems versus the general population, etc.) and context factors such as local legislation or country. However, variations also exist within the same populations and world regions. Results of the present study are context-dependent as well.

Although a significant proportion of patients here showed concerns about mental health risks of cannabis use, still two thirds of the sample did not mention negative consequences for mental health as a main reason for not using cannabis, and the items describing risks of RC use for mental or somatic health and cognitive functioning mostly showed moderate average agreement in both cohorts, markedly lower than “social distance” or “fear of negative attention”. The proportion of patients with a low score (<3) on the item addressing mental health risks was 25.9% (*n* = 37 out of 143) pre-RCL and 22.4% (*n* = 26 of 116) post-RCL. It therefore appears that a relevant subgroup tended to underestimate the potential harm from cannabis, which suggests room for further education on this matter. 

About ninety percent of the post-legalization group indicated that they would not consider using the newly introduced ways of obtaining cannabis (self-growing at home or becoming member of a cannabis social club, respectively). The idea to personally buy cannabis in licensed shops in the future was completely rejected at a somewhat lower rate (71.8%), but this rejection rate was higher than in the pre-RCL (58.7%) group. The cause for the increased rejection is not clear. Maybe the public discussion about RCL, which had included presentations of detrimental cannabis effects, had influenced the abstinence orientation of many patients. Moreover, the meaning of “would you buy in a specialized, licensed shop” had changed during RCL. Before RCL, the described shops could be interpreted as the only legal sources of recreational cannabis. Under the new legislation, other sources had already been permitted, and possession of cannabis for personal use was probably perceived as causing no trouble, regardless of its source. Therefore, the rejection of purchase in a licensed shop after RCL could have been caused by rejection of cannabis in general, but also by preference for other sources.

The lifetime prevalence of cannabis use was 64.3% pre-RCL and 65% post-RCL, the 12-month-prevalence was also similar, with 29.4% and 23.1%, respectively. According to a recent epidemiological survey of the German general population (18–64 years of age), the 12-month-prevalence of cannabis use was 9.8% [23]. The higher prevalence of cannabis use in the clinical sample might indicate that persons with mental disorders are at a higher risk of using cannabis. However, the elevated prevalence in the clinical samples might perhaps also partly due to the lower average age (35.4 and 35.2 years, compared to 44.9 years in the general population). 

Regarding patients’ attitudes and assumptions, the comparison of last year’s users versus non-users provides some insights: non-using patients differed markedly from users on items for “social distance” (mean difference of 2 points on a 7-point scale for some items), and showed higher levels of concern in most of the other topics covered by the questionnaire. The exceptions are “I noticed someone else becoming sluggish”, where recent users showed a significantly higher group mean than abstainers. It might be speculated that patients with cannabis use had more contact with other cannabis users, and therefore increasing the probability to observe such a negative consequence. With regard to spontaneously provided reasons for abstinence, the fear of becoming addicted (the second most frequent reason) was much more prevalent in recent nonusers than in users. Finally, in patients who had never used cannabis—or at least not during the previous year- the rejection rate of legal purchase was 68.9% before legalization and 80.2% afterwards. 

### Limitations

A major limitation of the present study is its repeated cross-sectional design, which prevents longitudinal observation of the same patients and modeling of true change. Additionally, monocentric recruitment prevents the assessment of how generalizable the results are beyond the present clinical context.

As mentioned previously, findings refer to patients with predominantly affective, anxiety, and personality disorders, but not to other important patient groups, especially those with schizophrenia or other forms of psychosis. Furthermore, because findings reflect a period shortly after RCL, future research is warranted to determine the long-term stability of these results. In addition, it is unclear how much the findings are influenced by a differential refusal rate, which was considerably higher in the post-RCL group. As mentioned above, this was partly because a number of patients in 2024/2025 participated in a concurrent study and a second participation felt like too much effort. We do not know whether the results can be generalized to those patients.

The questionnaire used in this study was self-designed and not internally or externally validated. It is unknown whether the use of other instruments measuring attitudes towards cannabis would modify the results. Because the study focused on attitudes towards cannabis shortly after RCL, no clinical or behavioural outcome measures were included. Furthermore, the assessment of cannabis use relied solely on self-report and is therefore of unknown reliability and validity; intentional or unintentional underreporting of cannabis use may have occurred. Finally, the clustering method (consensus-based clustering by two addiction specialists) has unknown reliability, and the results should thus be considered preliminary.

## 5. Conclusions

To conclude, among this selected sample of mental health patients without psychosis or substance use disorders, no marked differences in cannabis use frequency or attitudes toward cannabis were observed between groups investigated before versus shortly after RCL, except for the expected lower level of concerns about negative legal consequences of cannabis use after RCL. The risk assessment of cannabis use was complex, and the legal status of cannabis played only a minor role. Perceived social distance to cannabis and cannabis users was strongly endorsed by patients. There were concerns regarding mental health risks of cannabis use; however, it remains debatable whether the risk awareness was sufficient in this sample of patients with mental illness. Although large proportions of participants pre- and post-RCL endorsed regulated access to recreational cannabis use in principle, most of them, especially those with lifetime abstinence or no use during the past 12 months, showed no inclination to use such opportunities. Nevertheless, a relevant subgroup of patients in each cohort had used cannabis in the preceding year and/or was interested in legal purchase. In addition, it appears necessary to educate psychiatric patients, in particular, about the risks associated with recreational cannabis use for mental health [35].

## Figures and Tables

**Table 1:** Sociodemographic characteristics and main diagnoses.

	2022		2024/2025	
	*n*	%	*n*	%
*Gender*				
Male	54	37.8%	38	32.5%
Female	86	60.1%	78	66.7%
Other	3	2.1%	1	0.9%
*Age*				
Mean (Standard deviation)	35.4 (13.0)		35.2 (12.3)	
*School Education completed*				
None	2	1.3%	2	1.7%
Secondary school	65	45.5%	51	43.6%
High school diploma	71	49.7%	62	53.0%
Not specified	5	3.5%	2	1.7%
*Migrant background* ^1^				
Yes	37	25.9%	35	29.9%
No	104	72.7%	75	64.1%
Not specified	2	1.4%	7	6.0%
*Current employment*				
School/vocational training/university study	26	18.2%	20	17.1%
Employed	56	39.2%	46	39.4%
Not employed	47	32.9%	34	29.1%
Retired	4	2.8%	14	11.9%
Other	6	4.2%	3	2.6%
*Living situation*				
Living alone	63	44.1%	55	47.0%
Living with others	76	53.1%	58	49.6%
Residential facility	4	2.8%	3	2.6%
Homeless	0	0%	1	0.9%
*Relationship status*				
No relationship	82	57.3%	65	55.6%
Steady relationship, living apart	22	15.4	17	14.5%
Steady relationship, living together	39	27.3%	31	26.5%
Unknown	0	0.0%	4	3.4%
*Children in household*				
Yes	29	20.3%	14	12.0%
No	114	79.7%	103	88.0%
*Main ICD-10 diagnosis*				
F3× Mood [affective] disorder	122	85.3%	97	82.9%
F4× Neurotic, stress-related and somatoform disorder	9	6.3%	11	9.4%
F6× Disorder of adult personality and behavior	12	8.4%	9	7.7%

^1^ Patient or one of his/her parents foreign-born.

**Table 2:** History of psychotropic substance use.

	2022		2024/2025	
	*n*	%	*n*	%
*Lifetime recreational cannabis use*				
No use	51	35.7%	41	35.0%
Once or twice	25	17.5%	23	19.7%
3–10 times	20	14.0%	19	16.2%
11–50 times	22	15.4%	6	5.1%
>50 times	23	16.1%	13	11.1%
Frequency not specified	2	1.4%	15	12.8%
*Used cannabis at least once during last 12 months*				
Yes	42	29.4%	27	23.1%
No	101	70.6%	88	75.2%
Not answered	0	0%	2	1.7%
*Lifetime Substance Use (except cannabis and synthetic cannabinoids)*				
Alcohol, regular use ^1^	106	74.1%	71	61.7%
Any use of illicit or legally dubious substances	34	23.8%	32	27.4%
Regular use ^1^ of illicit or legally dubious substances	13	9.1%	15	12.8%
Non-medical use of prescribed medication ^2^	27	18.9%	33	28.2%
*Substance use last 30 days before admission*				
Alcohol	38	26.6%	27	23.1%
Illicit or legally dubious substances	3	2.1%	1	0.9%
*Tobacco and/or e-cigarette use*				
Current smoker	62	43.3%	45	38.5%
Previous smoker	21	14.7%	22	18.8%
Never smoker	60	42.0%	46	39.3%
Unknown	0	0%	4	3.4%

^1^ At least 10 times during one year. ^2^ Taken in higher doses than prescribed, or taken without prescription.

**Table 3:** Responses of both cohorts to the items concerning attitudes and beliefs with regard to cannabis on a 1-to-7-point scale.

Item	Group	*n*	Mean	SD	t	d.f.	*p* ^1^	Mean Difference	s.e. of Mean Difference	95% c.i. Lower Bound	95% c.i. Upper Bound
*Health and performance concerns*											
I could become mentally ill, or existing mental health issues could get worse.	2022	143	4.3	2.1	0.93	250.6	0.36	0.24	0.26	−0.28	0.75
	2024/2025	116	4.6	2.0							
I might start using hard drugs like heroin or cocaine.	2022	143	2.2	2.0	0.04	245.4	0.97	0.01	0.24	−0.47	0.49
	2024/2025	116	2.2	2.0							
I could get physically ill from consuming cannabis.	2022	143	3.3	2.1	0.25	238.9	0.80	0.07	0.27	−0.46	0.59
	2024/2025	115	3.4	2.2							
I could become sluggish and no longer manage my life properly.	2022	143	4.7	2.0	−0.89	248.9	0.38	−0.22	0.25	−0.70	0.27
	2024/2025	114	4.5	1.9							
I could become dependent on cannabis eventually.	2022	143	4.6	2.2	0.55	243.2	0.58	0.15	0.27	−0.39	0.69
	2024/2025	114	4.7	2.2							
I could not concentrate properly at school/university/work anymore and I quickly forget what I’ve learned.	2022	142	4.8	2.0	−0.35	235.7	0.73	−0.09	0.25	−0.58	0.40
	2024/2025	113	4.7	2.0							
As a non-smoker, I avoid smoking cannabis cigarettes (joints).	2022	73	4.5	2.5	2.22	131.8	0.03 *	0.89	0.41	0.10	1.69
	2024/2025	61	5.4	2.2							
*Legal and social problems*											
I would be afraid that the police might catch me consuming cannabis and then launch an investigation against me.	2022	143	4.5	2.4	−3.35	245.1	<0.001 *	−0.99	0.30	−1.58	−0.41
	2024/2025	116	3.6	2.4							
I’d be worried about getting in trouble at school/university/work if I got caught with cannabis.	2022	143	4.9	2.3	−1.49	247.1	0.14	−0.42	0.28	−0.97	0.13
	2024/2025	116	4.5	2.2							
I would be afraid of losing my driver’s license if I got caught with cannabis in traffic.	2022	98	5.6	2.0	−0.46	128.4	0.65	−0.15	0.32	−0.78	0.49
	2024/2025	62	5.4	2.0							
*Social Distance to Cannabis and Cannabis Users*											
As far as I know, no one in my circle of friends uses cannabis.	2022	143	4.0	2.6	−0.72	248.9	0.47	−0.23	0.32	−0.85	0.40
	2024/2025	116	3.8	2.5							
I do not want to have any contact with illegal drug scenes or dealers to obtain cannabis.	2022	143	5.9	2.0	−0.83	236.9	0.41	−0.21	0.25	−0.71	0.29
	2024/2025	114	5.7	2.0							
I wouldn’t even know how to obtain cannabis regularly.	2022	143	4.6	2.5	−1.12	244.7	0.23	−0.38	0.32	−1.01	0.24
	2024/2025	117	4.2	2.6							
Cannabis was never offered to me.	2022	143	2.8	2.5	−0.21	252.5	0.83	−0.06	0.30	−0.66	0.53
	2024/2025	117	2.7	2.4							
Cannabis wasn’t interesting to me as a teenager and young adult because nobody in my circle of friends used it.	2022	143	3.8	2.6	0.26	252.2	0.80	0.08	0.32	−0.55	0.71
	2024/2025	117	3.8	2.5							
*Rejection of Drugs*											
I think people should live their lives without the influence of drugs.	2022	143	5.2	2.0	1.40	256.4	0.16	0.32	0.24	−0.14	0.79
	2024/2025	116	5.5	1.7							
I do not take drugs that are prohibited in our country.	2022	143	5.6	2.2	0.71	246.6	0.48	0.19	0.27	−0.34	0.73
	2024/2025	116	5.8	2.2							
*Educational Influences*											
I was kept from using cannabis by drug education at school and in the media.	2022	143	2.9	2.2	0.22	245.0	0.83	0.06	0.28	−0.49	0.61
	2024/2025	116	2.9	2.3							
I was kept from using cannabis by my parents’ clear disapproval.	2022	143	3.3	2.3	0.04	244.2	0.97	0.01	0.29	−0.56	0.58
	2024/2025	116	3.3	2.3							
*Observation of negative consequences*											
I noticed how someone else (friend, acquaintance, classmate, colleague, family member) became sluggish from using cannabis and was no longer able to handle their tasks.	2022	143	4.0	2.5	0.31	243.5	0.76	0.10	0.31	−0.52	0.71
	2024/2025	114	4.1	2.5							
I witnessed someone else (friend, acquaintance, classmate, colleague, family member) get into trouble with the police because of cannabis use.	2022	143	3.7	2.7	−1.34	251.1	0.18	−0.43	0.33	−1.08	0.21
	2024/2025	115	3.3	2.5							
I noticed someone else (friend, acquaintance, classmate, co-worker, family member) became psychotic from cannabis use (e.g., with hallucinations, delusions).	2022	143	2.7	2.4	0.50	244.1	0.62	0.15	0.30	−0.44	0.74
	2024/2025	115	2.9	2.4							

^1^ Welch *t*-test. * Statistically significant on the 0.05 level.

**Table 4:** Attitudes towards legal procurement of cannabis. Percentages are based on total number of valid responses.

	2022		2024/2025			
	*n*	%	*n*	%	*p* ^1^	Somer’s D (95% c.i.)
*Attitude towards the general possibility of a legal purchase in licensed shops*						
Endorse	71	49.7%	55	47.4%	0.71	0.03 [−0.06; 0.12]
Undecided	30	21.0%	27	23.3%		
Reject	42	29.4%	34	29.3%		
Not answered	0		1			
*Would buy him/herself cannabis in licensed shops*						
Certainly	10	7.0%	7	6.0%	0.02	0.14 [0.03; 0.25]
Probably	18	12.6%	5	4.3%		
Perhaps	31	21.7%	21	17.9%		
No	84	58.7%	84	71.8%		
*Will obtain cannabis legally through cannabis social club*						
Yes			3	2.6%		
Perhaps			8	6.8%		
No			106	90.6%		
*Will grow cannabis legally myself*						
Yes			1	0.9%		
Perhaps			11	9.4%		
No			105	89.7%		

^1^ Mann–Whitney U-test.

**Table 5:** Proportion of participants who stated the respective reason for abstinence, by cannabis use. Percentages refer to columns.

Important Reasons for Not Using Cannabis	Cannabis Use Within the Last 12 Months			Odds Ratio [95% c.i.]
	Yes (*n* = 69)	No (*n* = 189)	*p* ^1^	
Negative impact on mental health	37.7% (*n* = 26)	27.0% (*n* = 51)	0.10	1.64 [0.91; 2.9]
Become dependent or addicted	5.8% (*n* = 4)	23.8% (*n* = 45)	<0.001	0.20 [0.07; 0.57]
Not interested, no need	8.3% (*n* = 6)	14.8% (*n* = 28)	0.20	0.55 [0.22; 1.39]
Illegal status, fear of negative consequences	10.1% (*n* = 7)	5.8% (*n* = 11)	0.23	1.83 [0.68; 4.92]
Negative impact on physical health	1.4% (*n* = 1)	12.2% (*n* = 23)	0.009	0.11 [0.01; 0.80]
Impairment of social and everyday functioning	18.8% (*n* = 13)	9.0% (*n* = 17)	0.03	2.35 [1.07; 5.14]
Loss of control, change in consciousness	7.2% (*n* = 5)	12.2% (*n* = 23)	0.26	0.56 [0.21; 1.55]

^1^ Chi square test.

## Data Availability

Data are not publicly available due to privacy restrictions. Data can be made available upon request to the authors (send to: M.S.).

## Supplementary Materials

The following supporting information can be downloaded at: https://www.mdpi.com/article/10.3390/brainsci16070730/s1, Supplementary S1: Interview form, German version; S2: Interview form, English translation; S3: Output of the multiple linear regression analyses; S4: Comparison of responses to attitude items between recent cannabis users and nonusers.

## Abbreviations

The following abbreviations are used in this manuscript:

c.i.	Confidence interval
RC	Recreational cannabis
RCL	Legalization of recreational cannabis
s.e.	Standard error
