# Supplementary material for: Attitudes of Patients with Non-Psychotic Mental Disorders Towards Cannabis After Its Legalization—Comparison with Patients Before Legalization
Source: Brain Sci. 2026 Jul 11;16(7):730. doi: 10.3390/brainsci16070730 (PMC13406209; doi:10.3390/brainsci16070730)
Supplement: Supplementary file 1 [file brainsci-16-00730-s001.zip › S1 Interview_Attitudes_Towards_Cannabis_Versions_2022_and_2024_German.pdf]

Deckblatt

Untersuchung zur Einstellung zur Einnahme von Cannabis („Haschisch", „Marihuana")

Erhebungsdatum ________________________

Station / Ambulanz ________________________________

Patient/innen-Code: ___________________

ICD-Diagnosen (bei Behandlungsende):

____

____

Gehört zu den Diagnosen eine cannabisbezogene Störung (ICD-10 F12.1 bzw. F12.2)?

❑ nein

❑ ja **=> Ausschlusskriterium**

Untersuchung zu Einstellungen gegenüber der Einnahme von Cannabis („Haschisch“, „Marihuana“)

Allgemeine Angaben

Alter: ______

Geschlecht: m ❑ w ❑ d ❑

Wegen welcher psychischen Erkrankung (einschließlich Suchterkrankungen) sind Sie zurzeit in Behandlung im LVR-Klinikum Essen (gegebenenfalls auch mehrere Erkrankungen)?

Ihr Geburtsland: ____________

Geburtsland Mutter / Vater:

____________________ / ____________________

Bildungsabschluss:

- ❑ Keiner
- ❑ Hauptschulabschluss
- ❑ Mittlere Reife, Fachoberschulreife, Realschulabschluss
- ❑ Fachabitur oder Abitur
- ❑ Studium abgeschlossen
- ❑ Sonstiger: ______________________

Tätigkeit in den 30 Tagen vor Aufnahme:

- ❑ Auszubildende(r)/Schüler(in)/Student(in)
- ❑ Vollzeit erwerbstätig (ggf. aktuell arbeitsunfähig erkrankt)
- ❑ Teilzeit erwerbstätig (ggf. aktuell arbeitsunfähig erkrankt)
- ❑ erwerbslos
- ❑ Sonstiges:______________________

Wohnsituation vor Aufnahme:

- ❑ Allein in eigener Wohnung
- ❑ Mit anderen in eigener Wohnung
- ❑ Wohneinrichtung, Heim
- ❑ Wohnungslos
- ❑ Sonstiges:______________________

Beziehungsstatus:

- ❑ alleinstehend
- ❑ feste Beziehung, getrennt lebend
- ❑ feste Beziehung, zusammenlebend

Von Ihnen zu versorgende Kinder im Haushalt:

❑ ja ❑ nein

Haben Sie Erfahrungen mit dem Konsum folgender Suchtmittel bzw. Drogen? (zutreffendes bitte ankreuzen)

	Mindestens einmal im Leben konsumiert	Schonmal regelmäßig konsumiert (mindestens 10mal innerhalb eines Jahres)	In den letzten 30 Tagen vor jetziger stationärer Aufnahme konsumiert
Alkohol	❑	❑	❑
Tabak	❑	❑	❑
E-Zigaretten	❑	❑	❑
Amphetamine, Methamphetamin	❑	❑	❑
Synthetische Cannabinoide (z.B. „Spice")	❑	❑	❑
Ecstasy	❑	❑	❑
Heroin	❑	❑	❑
Kokain	❑	❑	❑
LSD	❑	❑	❑
Eine andere Substanz (z.B. Schnüffelstoffe, psychedelische Pilze, „Naturdrogen" usw.)?<br>Name: ______________	❑	❑	❑
Eine weitere Substanz?<br>Name:_______________	❑	❑	❑

Arzneimittel-Fehlgebrauch:

Haben Sie folgende Medikamente jemals in höherer Dosis eingenommen als vom Arzt verordnet? Oder sich diese Medikamente ohne Rezept besorgt und eingenommen?

❑ ADHS-Medikamente wie Methylphenidat (z.B. Ritalin, Medikinet, Concerta), Dexamfetamin (z.B. Attentin), Lisdexamfetamin

❑ Gabapentin, Pregabalin (z.B. Lyrica)

❑ Benzodiazepine oder Z-Substanzen (z.B. Zopiclon, Zolpidem)

❑ Opiathaltige Schmerzmittel bzw. Hustensäfte (z.B. Tilidin, Tramadol, Codein)

❑ Andere Medikamente ____________________

Was glauben Sie könnte passieren, wenn Sie Cannabis zu sich nehmen?

Ich könnte psychisch krank werden oder bestehende psychische Erkrankungen könnten schlimmer werden.

❑	❑	❑	❑	❑	❑	❑

Trifft gar nicht zu — Trifft sehr zu

Es könnte sein, dass ich auch zu harten Drogen wie Heroin oder Kokain greife.

❑	❑	❑	❑	❑	❑	❑

Trifft gar nicht zu — Trifft sehr zu

Ich könnte durch den Konsum von Cannabis körperlich krank werden.

❑	❑	❑	❑	❑	❑	❑

Trifft gar nicht zu — Trifft sehr zu

Ich könnte träge werden und mein Leben nicht mehr richtig bewältigen.

❑	❑	❑	❑	❑	❑	❑

Trifft gar nicht zu — Trifft sehr zu

Ich könnte von Cannabis irgendwann abhängig werden.

❑	❑	❑	❑	❑	❑	❑

Trifft gar nicht zu — Trifft sehr zu

Ich könnte mich in der Schule / im Studium / bei der Arbeit nicht mehr richtig konzentrieren oder Lernstoff schnell wieder vergessen.

❑	❑	❑	❑	❑	❑	❑

Trifft gar nicht zu — Trifft sehr zu

Ich hätte Angst, dass die Polizei mich beim Cannabiskonsum erwischen könnte, und dann gegen mich ermittelt wird.

❑	❑	❑	❑	❑	❑	❑

Trifft gar nicht zu — Trifft sehr zu

Ich hätte Angst, dass ich in der Schule / an der Universität / an meinem Arbeitsplatz Ärger bekomme, wenn ich mit Cannabis auffalle.

❑	❑	❑	❑	❑	❑	❑

Trifft gar nicht zu — Trifft sehr zu

Ich hätte Angst, meinen Führerschein zu verlieren, wenn ich mit Cannabiskonsum im Verkehr auffalle.

❑	❑	❑	❑	❑	❑	❑

Trifft gar nicht zu — Trifft sehr zu

Wie sieht es mit Cannabiskonsum allgemein in Ihrem Leben aus?

Soweit ich weiß, wird in meinem Bekanntenkreis kein Cannabis konsumiert.

❑	❑	❑	❑	❑	❑	❑

Trifft gar nicht zu — Trifft sehr zu

Ich will keinen Kontakt mit illegalen Drogenszenen oder Dealern haben, um mir Cannabis zu beschaffen.

❑	❑	❑	❑	❑	❑	❑

Trifft gar nicht zu — Trifft sehr zu

Ich wüsste gar nicht, wie man regelmäßig an Cannabis kommt.

❑	❑	❑	❑	❑	❑	❑

Trifft gar nicht zu — Trifft sehr zu

Ich finde, man sollte sein Leben ohne den Einfluss von Drogen führen.

❑	❑	❑	❑	❑	❑	❑

Trifft gar nicht zu — Trifft sehr zu

Ich nehme keine Drogen, die in unserem Land verboten sind.

❑	❑	❑	❑	❑	❑	❑

Trifft gar nicht zu — Trifft sehr zu

Mir wurde nie Cannabis angeboten.

❑	❑	❑	❑	❑	❑	❑

Trifft gar nicht zu — Trifft sehr zu

Ich wurde durch die Drogenaufklärung in der Schule und den Medien vom Konsum von Cannabis abgehalten.

❑	❑	❑	❑	❑	❑	❑

Trifft gar nicht zu — Trifft sehr zu

Ich wurde durch die eindeutige Ablehnung meiner Eltern vom Cannabiskonsum abgehalten.

❑	❑	❑	❑	❑	❑	❑

Trifft gar nicht zu — Trifft sehr zu

Ich habe mitbekommen, wie jemand anderes (Freund/in, Bekannter, Mitschülerin/Mitschüler, Arbeitskollege, Familienmitglied) durch Cannabiskonsum träge geworden ist und seine Aufgaben nicht mehr bewältigen konnte.

❑	❑	❑	❑	❑	❑	❑

Trifft gar nicht zu — Trifft sehr zu

Cannabis war für mich als Jugendlicher und junger Erwachsener nicht interessant, da in meinem Freundeskreis niemand Cannabis konsumiert hat.

❑	❑	❑	❑	❑	❑	❑

Trifft gar nicht zu — Trifft sehr zu

Ich habe mitbekommen, wie jemand anderes (Freund/in, Bekannter, Mitschüler/in, Arbeitskollege/-kollegin, Familienmitglied) durch Cannabiskonsum Ärger mit der Polizei bekommen hat.

❑	❑	❑	❑	❑	❑	❑

Trifft gar nicht zu — Trifft sehr zu

Ich habe mitbekommen, wie jemand anderes (Freund/in, Bekannter, Mitschülerin/Mitschüler, Arbeitskollege, Familienmitglied) durch Cannabiskonsum psychotisch wurde (z.B. mit Halluzinationen, Wahnvorstellungen)

❑	❑	❑	❑	❑	❑	❑

Trifft gar nicht zu — Trifft sehr zu

Frage für Nichtraucher:

Als Nichtraucher lehne ich den Konsum von Cannabiszigaretten (Joints) ab.

❑	❑	❑	❑	❑	❑	❑

Trifft gar nicht zu — Trifft sehr zu

Haben Sie in Ihrem Leben schon mal Cannabis konsumiert?

❑ nein => bitte weiterblättern zur letzten Seite

❑ ja

In welchem Alter (oder in welchem Jahr) haben Sie zum ersten Mal Cannabis konsumiert? __________________

In welchem Alter (oder in welchem Jahr) zum letzten Mal? __________________

An wie vielen Tagen haben Sie etwa im Leben Cannabis konsumiert? ____Tage

Gab es eine Zeit, in der Sie Cannabis **regelmäßig** (mindestens 10mal in einem Zeitraum von 12 Monaten) konsumiert haben?

❑ nein ❑ ja

Haben Sie in den letzten 12 Monaten Cannabis **mindestens einmal** konsumiert?

❑ nein ❑ ja

Wie haben Sie Ihr Cannabis erhalten (Mehrfachnennungen möglich)?

- ❑ Gekauft
- ❑ Geschenkt bekommen
- ❑ Mitkonsumiert, was andere mitgebracht hatten
- ❑ Sonstiges _______________________

Haben Sie jemals medizinisches Cannabis verschrieben bekommen?

❑ nein ❑ ja, in folgenden Zeiträumen _______________

Wie fanden Sie die Wirkung von Cannabis?

Ich fand die Wirkung von Cannabis langweilig.

❑	❑	❑	❑	❑	❑	❑

Trifft gar nicht zu — Trifft sehr zu

Ich mochte die Wirkung.

❑	❑	❑	❑	❑	❑	❑

Trifft gar nicht zu — Trifft sehr zu

Ich fühlte mich körperlich unwohl, als ich Cannabis konsumierte.

❑	❑	❑	❑	❑	❑	❑

Trifft gar nicht zu — Trifft sehr zu

Der Konsum hat mich entspannt.

❑	❑	❑	❑	❑	❑	❑

Trifft gar nicht zu — Trifft sehr zu

Ich war mindestens einmal psychotisch (Verfolgungserleben, Sinnestäuschungen etc.) unter Cannabis.

❑	❑	❑	❑	❑	❑	❑

Trifft gar nicht zu — Trifft sehr zu

Ich fühlte mich unter dem Einfluss von Cannabis weniger ängstlich

❑	❑	❑	❑	❑	❑	❑

Trifft gar nicht zu — Trifft sehr zu

Ich habe mich durch den Cannabiskonsum in meiner psychischen Gesundheit beeinträchtigt gefühlt.

❑	❑	❑	❑	❑	❑	❑

Trifft gar nicht zu — Trifft sehr zu

Unter der Wirkung von Cannabis war ich in guter Stimmung

❑	❑	❑	❑	❑	❑	❑

Trifft gar nicht zu — Trifft sehr zu

Letzte Seite [Version 2022]

Spontan gesagt, was ist für Sie der wichtigste Grund oder sind die wichtigsten Gründe dafür, kein Cannabis zu konsumieren?

Es ist vorgesehen, dass zukünftig der Besitz und Erwerb von Cannabis zum Freizeitkonsum straffrei ist und Cannabis legal in speziellen Verkaufsstellen erworben werden kann. Würden Sie dies befürworten?

- ❑ Ja, das würde ich befürworten
- ❑ Nein, ich bin dagegen
- ❑ Ich bin in der Frage unentschieden

Würden Sie dort Cannabis kaufen?

- ❑ nein
- ❑ vielleicht
- ❑ wahrscheinlich
- ❑ sicher

Vielen Dank für Ihre Mithilfe

Letzte Seite [Version 2024/2025]

Spontan gesagt, was ist für Sie der wichtigste Grund oder sind die wichtigsten Gründe dafür, kein Cannabis zu konsumieren?

____

Nach der neuen Gesetzeslage darf Cannabis zum Eigenbedarf selbst angebaut werden. Werden Sie einen solchen Eigenanbau betreiben?

- ❑ nein
- ❑ vielleicht
- ❑ wahrscheinlich
- ❑ sicher

Nach der neuen Gesetzeslage kann man regelmäßig zahlendes Mitglied in einer Cannabis-Anbauvereinigung werden, um Cannabis legal zu erhalten. Werden Sie auf diese Art Cannabis beziehen?

- ❑ nein
- ❑ vielleicht
- ❑ wahrscheinlich
- ❑ sicher

Es ist in Zukunft vorgesehen, dass Cannabis legal in speziellen Verkaufsstellen erworben werden kann. Würden Sie dies befürworten?

- ❑ Ja, das würde ich befürworten
- ❑ Nein, ich bin dagegen
- ❑ Ich bin in der Frage unentschieden

Würden Sie dort Cannabis kaufen?

- ❑ nein
- ❑ vielleicht
- ❑ wahrscheinlich
- ❑ sicher

Vielen Dank für Ihre Mithilfe
