# Supplementary material for: Attitudes of Patients with Non-Psychotic Mental Disorders Towards Cannabis After Its Legalization—Comparison with Patients Before Legalization
Source: Brain Sci. 2026 Jul 11;16(7):730. doi: 10.3390/brainsci16070730 (PMC13406209; doi:10.3390/brainsci16070730)
Supplement: Supplementary file 1 [file brainsci-16-00730-s001.zip › S2 Interview_Attitudes_towards_Cannabis_English_Translation_June16_2026.pdf]

# Cover Sheet

# Investigation about Attitudes towards Consumption of Cannabis (“Hashish“, ”Marijuana“)

Date ________________________

Department ________________________________

Patient code: ___________________

ICD diagnoses (at discharge):

______________________________________________________________

______________________________________________________________

**Was a Cannabis-related disorder (ICD-10 F12.1 or F12.2) diagnosed?**

❑ no

❑ yes **=> Exclusion**

# Investigation about Attitudes towards Consumption of Cannabis (“Haschisch“, ”Marijuana“)

## General Information

Age: ______

Gender: m ❑ f ❑ d ❑

Which mental illness (including addiction) is the reason for your current treatment in this hospital (you can indicate more than one, if applicable)?

___________________________________________

Your country of birth: ____________

Country of birth: Mother / Father:

___________________ / ___________________

Education completed:

- ❑ None
- ❑ Secondary school leaving certificate
- ❑ Comprehensive school leaving certificate
- ❑ High school diploma
- ❑ University study
- ❑ Other: _____________________

Occupation during the last 30 days before admission:

- ❑ In training/school/university

- ❑ Employed full-time (may be currently unable to work because of illness)
- ❑ Employed part-time (may be currently unable to work because of illness)
- ❑ Unemployed
- ❑ Other:________________________

Housing situation before admission:

- ❑ live by myself in my own flat
- ❑ live with others, in a flat
- ❑ Assisted living facility
- ❑ Homeless
- ❑ Other:________________________

Relationship status:

- ❑ single
- ❑ steady relationship, living apart
- ❑ steady relationship, living together

Living with children you have to take care of:

❑ yes ❑ no

**Did you consume the following drugs? (please check the relevant boxes)**

| | Consumed at least once during lifetime | Consumed regularly (at least 10 times within one year) | Consumed within the last 30 days before admission |
|---|---|---|---|
| Alcohol | ❑ | ❑ | ❑ |
| Tobacco | ❑ | ❑ | ❑ |
| E-cigarettes | ❑ | ❑ | ❑ |
| Amphetamines, Methamphetamine | ❑ | ❑ | ❑ |
| Synthetic Cannabinoids (e.g. “Spice“) | ❑ | ❑ | ❑ |
| Ecstasy | ❑ | ❑ | ❑ |
| Heroin | ❑ | ❑ | ❑ |
| Cocaine | ❑ | ❑ | ❑ |
| LSD | ❑ | ❑ | ❑ |
| Other substance (e.g. inahalants, psychoactive mushrooms, „natural drugs “ etc.)?<br>Name: ______________ | ❑ | ❑ | ❑ |
| One more substance?<br>Name:_______________ | ❑ | ❑ | ❑ |

**Misuse of medication:**

**Did you ever take one of the following medications in higher dose than-prescribed by a physician? Or, did you take them without prescription?**

❑ ADHS-medication, like Methylphenidate (Ritalin, Concerta), Dextroampheta-mine (e.g. Dexedrine, Zenzedi,), Lisdexamfetamine (Vivanse, Evanse)

❑ Gabapentin, Pregabalin (e.g. Axalid, Lyrica)

❑ Benzodiazepines, Z-substances (e.g. Zopiclon, Zolpidem)

❑ Opioid analgesics (e.g.. Tilidine, Valorone, Tramadol/Tramal, Codein)

❑ Other medications ____________________

## What do you think could happen if you consume cannabis?

I could become mentally ill, or existing mental health issues could get worse.

| | | | | | | |
|---|---|---|---|---|---|---|
| ❑ | ❑ | ❑ | ❑ | ❑ | ❑ | ❑ |

Does not apply at all — Applies very much

I might start using hard drugs like heroin or cocaine.

| | | | | | | |
|---|---|---|---|---|---|---|
| ❑ | ❑ | ❑ | ❑ | ❑ | ❑ | ❑ |

Does not apply at all — Applies very much

"I could get physically ill from consuming cannabis.

| | | | | | | |
|---|---|---|---|---|---|---|
| ❑ | ❑ | ❑ | ❑ | ❑ | ❑ | ❑ |

Does not apply at all — Applies very much

I could become sluggish and no longer manage my life properly.

| | | | | | | |
|---|---|---|---|---|---|---|
| ❑ | ❑ | ❑ | ❑ | ❑ | ❑ | ❑ |

Does not apply at all — Applies very much

I could become dependent on cannabis eventually.

| | | | | | | |
|---|---|---|---|---|---|---|
| ❑ | ❑ | ❑ | ❑ | ❑ | ❑ | ❑ |

Does not apply at all — Applies very much

I could not concentrate properly at school/university/work anymore and I quickly forget what I've learned.

| ❑ | ❑ | ❑ | ❑ | ❑ | ❑ | ❑ |
|---|---|---|---|---|---|---|

Does not apply at all | Applies very much

I would be afraid that the police might catch me consuming cannabis and then launch an investigation against me.

| ❑ | ❑ | ❑ | ❑ | ❑ | ❑ | ❑ |
|---|---|---|---|---|---|---|

Does not apply at all | Applies very much

I'd be worried about getting in trouble at school / university / work if I got caught with cannabis.

| ❑ | ❑ | ❑ | ❑ | ❑ | ❑ | ❑ |
|---|---|---|---|---|---|---|

Does not apply at all | Applies very much

I would be afraid of losing my driver's license if I got caught with cannabis in traffic.

| ❑ | ❑ | ❑ | ❑ | ❑ | ❑ | ❑ |
|---|---|---|---|---|---|---|

Does not apply at all | Applies very much

## What is your general experience with cannabis use?

As far as I know, no one in my circle of friends uses cannabis.

| ❑ | ❑ | ❑ | ❑ | ❑ | ❑ | ❑ |
|---|---|---|---|---|---|---|

Does not apply at all | Applies very much

I do not want to have any contact with illegal drug scenes or dealers to obtain cannabis.

| ❑ | ❑ | ❑ | ❑ | ❑ | ❑ | ❑ |
|---|---|---|---|---|---|---|

Does not apply at all | Applies very much

"I wouldn't even know how to get cannabis regularly.

| ❑ | ❑ | ❑ | ❑ | ❑ | ❑ | ❑ |
|---|---|---|---|---|---|---|

Does not apply at all | Applies very much

I think people should live their lives without the influence of drugs.

| | | | | | | |
|---|---|---|---|---|---|---|
| ❑ | ❑ | ❑ | ❑ | ❑ | ❑ | ❑ |

Does not apply at all | Applies very much

I do not take drugs that are prohibited in our country.

| | | | | | | |
|---|---|---|---|---|---|---|
| ❑ | ❑ | ❑ | ❑ | ❑ | ❑ | ❑ |

Does not apply at all | Applies very much

Cannabis was never offered to me.

| | | | | | | |
|---|---|---|---|---|---|---|
| ❑ | ❑ | ❑ | ❑ | ❑ | ❑ | ❑ |

Does not apply at all | Applies very much

I was kept from using cannabis by drug education at school and in the media.

| | | | | | | |
|---|---|---|---|---|---|---|
| ❑ | ❑ | ❑ | ❑ | ❑ | ❑ | ❑ |

Does not apply at all | Applies very much

I was kept from using cannabis by my parents' clear disapproval.

| | | | | | | |
|---|---|---|---|---|---|---|
| ❑ | ❑ | ❑ | ❑ | ❑ | ❑ | ❑ |

Does not apply at all | Applies very much

I noticed how someone else (friend, acquaintance, classmate, colleague, family member) became sluggish from using cannabis and was no longer able to handle their tasks.

| | | | | | | |
|---|---|---|---|---|---|---|
| ❑ | ❑ | ❑ | ❑ | ❑ | ❑ | ❑ |

Does not apply at all | Applies very much

Cannabis wasn't interesting to me as a teenager and young adult because no-body in my circle of friends used it.

| | | | | | | |
|---|---|---|---|---|---|---|
| ❑ | ❑ | ❑ | ❑ | ❑ | ❑ | ❑ |

Does not apply at all | Applies very much

I witnessed someone else (friend, acquaintance, classmate, colleague, family member) get into trouble with the police because of cannabis use.

| | | | | | | |
|---|---|---|---|---|---|---|
| ❑ | ❑ | ❑ | ❑ | ❑ | ❑ | ❑ |

Does not apply at all — Applies very much

I noticed someone else (friend, acquaintance, classmate, coworker, family member) became psychotic from cannabis use (e.g., with hallucinations, delusions).

| | | | | | | |
|---|---|---|---|---|---|---|
| ❑ | ❑ | ❑ | ❑ | ❑ | ❑ | ❑ |

Does not apply at all — Applies very much

*Question for non-smokers:*

As a non-smoker, I avoid smoking cannabis cigarettes (joints).

| | | | | | | |
|---|---|---|---|---|---|---|
| ❑ | ❑ | ❑ | ❑ | ❑ | ❑ | ❑ |

Does not apply at all — Applies very much

**Have you ever consumed cannabis in your life?**

❑ no => please go to the last page

❑ yes

At which age (or in which year) did you consume cannabis fort the first time?

______________

At which age (or in which year) did you consume the last time? ______________

How many day in total did you ever consume cannabis? ______ days

Was there at time you consumed cannabis **regularly** (at least 10 times within 12 months)?

❑ no ❑ yes

Haben Sie in den letzten 12 Monaten Cannabis **mindestens einmal** konsumiert?

❑ no ❑ yes

**How did you obtain cannabis (multiple answers possible)?**

- ❑ Purchased it
- ❑ Received it as a gift
- ❑ Joined in when others started smoking their own cannabis
- ❑ Other ______________________

Were you ever prescribed medical cannabis?

❑ no ❑ yes, during these periods: ________________________

**How did you like the effects of cannabis?**

I found the effects of cannabis boring.

| ❑ | ❑ | ❑ | ❑ | ❑ | ❑ | ❑ |
|---|---|---|---|---|---|---|
| Does not apply at all | | | | | | Applies very much |

I liked the effect.

| ❑ | ❑ | ❑ | ❑ | ❑ | ❑ | ❑ |
|---|---|---|---|---|---|---|

Does not apply at all — Applies very much

I fehlt physically unwell when I consumed cannabis.

| ❑ | ❑ | ❑ | ❑ | ❑ | ❑ | ❑ |
|---|---|---|---|---|---|---|

Does not apply at all — Applies very much

Consumption of cannabis relaxed me.

| ❑ | ❑ | ❑ | ❑ | ❑ | ❑ | ❑ |
|---|---|---|---|---|---|---|

Does not apply at all — Applies very much

I became psychotic (paranoia, hallucinations etc.) at least once under the influence of cannabis.

| ❑ | ❑ | ❑ | ❑ | ❑ | ❑ | ❑ |
|---|---|---|---|---|---|---|

Does not apply at all — Applies very much

I felt less anxious under the influence of cannabis.

| ❑ | ❑ | ❑ | ❑ | ❑ | ❑ | ❑ |
|---|---|---|---|---|---|---|

Does not apply at all — Applies very much

My mental health was impaired.

| ❑ | ❑ | ❑ | ❑ | ❑ | ❑ | ❑ |
|---|---|---|---|---|---|---|

Does not apply at all — Applies very much

I was in good mood under the influence of cannabis.

| ❑ | ❑ | ❑ | ❑ | ❑ | ❑ | ❑ |
|---|---|---|---|---|---|---|

Does not apply at all — Applies very much

**Last page [Version 2022]**

What was the most important reason or were the most important reasons for you not to take cannabis?

---

It is intended that possession and acquisition of cannabis for recreational use are exempted from punishment, and that cannabis can be purchased in specialized shops. Would you approve this?

- ❑ Yes, I would approve this
- ❑ No, I am against it
- ❑ I am undecided

Would you buy cannabis there?

- ❑ no
- ❑ perhaps
- ❑ probably
- ❑ certainly

***Thank you for your cooperation!***

**Last page [Version 2024/2025]**

What was the most important reason or were the most important reasons for you not to take cannabis?

---

According to the new legislation, cannabis can be home cultivated for own consumption. Will you engage in such home cultivation?

- ❑ no
- ❑ perhaps
- ❑ probably
- ❑ certainly

According to the new legislation, one can become regularly paying member of a cannabis cultivation society, to obtain cannabis legally. Will you obtain cannabis this way??

- ❑ no
- ❑ perhaps
- ❑ probably
- ❑ certainly

It is intended that cannabis can be purchased in specialized shops. Would you approve this?

- ❑ Yes, I would approve this
- ❑ No, I am against it
- ❑ I am undecided

Would you buy cannabis there?

- ❑ no
- ❑ perhaps
- ❑ probably
- ❑ certainly

***Thank you for your cooperation!***
