# Supplementary material for: Attitudes of Patients with Non-Psychotic Mental Disorders Towards Cannabis After Its Legalization—Comparison with Patients Before Legalization
Source: Brain Sci. 2026 Jul 11;16(7):730. doi: 10.3390/brainsci16070730 (PMC13406209; doi:10.3390/brainsci16070730)
Supplement: Supplementary file 1 [file brainsci-16-00730-s001.zip › S3 Linear regression analyses of attitude items on patient characteristics.pdf]

Supplement S3: Linear regressions of attitude items on sociodemographic and clinical variables.

Dependent Variable: Could become mentally ill

Model (n=258)	Unstandardized Coefficients B	Unstandardized Coefficients Std. Error	95% confidence interval Lower limit	95% confidence interval Upper limit	Beta	t	p
(Constant)	4.061	0.531	3.020	5.101		7.649	0.0000
Group	0.149	0.257	-0.354	0.653	0.036	0.581	0.5618
Main diagnosis personality disorder (0="no", 1 = "yes")	0.268	0.474	-0.661	1.197	0.035	0.566	0.5720
Main diagnosis F4 (0="no", 1 = "yes")	0.379	0.496	-0.593	1.350	0.048	0.764	0.4455
Age in years	0.006	0.011	-0.016	0.028	0.038	0.555	0.5797
Gender (0=female, 1=male)	-0.126	0.226	-0.568	0.317	-0.035	-0.556	0.5785
Migrant background (0="no", 1 = "yes")	-0.034	0.295	-0.613	0.545	-0.007	-0.114	0.9090
School education > 10 years (0="no", 1 = "yes")	0.720	0.264	0.202	1.237	0.173	2.726	0.0069
Employed full time ((0="no", 1 = "yes")	-0.485	0.288	-1.049	0.080	-0.107	-1.683	0.0937
Current smoker ((0="no", 1 = "yes")	0.291	0.288	-0.274	0.856	0.069	1.010	0.3134
Lifetime Cannabis use (0="no", 1 = "yes")	-0.034	0.321	-0.664	0.596	-0.008	-0.105	0.9163
Cannabis use last 12 months (0="no", 1 = "yes")	-0.880	0.348	-1.563	-0.198	-0.187	-2.528	0.0121

Dependent Variable: Could resort to "hard drugs"

Model (n=258)	Unstandardized Coefficients B	Std. Error	95% confidence interval Lower limit	Upper limit	Beta	t	p
(Constant)	2.565	0.503	1.580	3.550		5.103	0.0000
Group	-0.062	0.243	-0.539	0.415	-0.016	-0.254	0.7994
Main diagnosis personality disorder (0="no", 1 = "yes")	0.319	0.449	-0.560	1.199	0.045	0.711	0.4775
Main diagnosis F4 (0="no", 1 = "yes")	-0.468	0.469	-1.388	0.452	-0.063	-0.996	0.3202
Age in years	-0.001	0.011	-0.022	0.020	-0.009	-0.126	0.8996
Gender (0=female, 1=male)	-0.327	0.214	-0.746	0.092	-0.097	-1.528	0.1279
Migrant background (0="no", 1 = "yes")	0.617	0.280	0.068	1.165	0.141	2.205	0.0284
School education > 10 years (0="no", 1 = "yes")	0.060	0.250	-0.430	0.550	0.015	0.240	0.8104
Employed full time ((0="no", 1 = "yes")	0.095	0.273	-0.440	0.629	0.022	0.347	0.7286
Current smoker ((0="no", 1 = "yes")	-0.036	0.273	-0.571	0.499	-0.009	-0.133	0.8946
Lifetime Cannabis use (0="no", 1 = "yes")	-0.290	0.304	-0.887	0.306	-0.071	-0.954	0.3412
Cannabis use last 12 months (0="no", 1 = "yes")	-0.534	0.330	-1.180	0.113	-0.121	-1.618	0.1068

Dependent Variable: Could become physically ill

Model (n=257)	Unstandardized Coefficients B	Std. Error	95% confidence interval Lower limit	Upper limit	Beta	t	p
(Constant)	4.101	0.525	3.071	5.131		7.806	0.0000
Group	-0.047	0.255	-0.547	0.452	-0.011	-0.186	0.8524
Main diagnosis personality disorder (0="no", 1 = "yes")	0.278	0.469	-0.641	1.197	0.036	0.592	0.5542
Main diagnosis F4 (0="no", 1 = "yes")	0.488	0.491	-0.474	1.450	0.060	0.994	0.3214

Age in years	0.004	0.011	-0.018	0.026	0.026	0.385	0.7004
Gender (0=female, 1=male)	-0.380	0.224	-0.818	0.059	-0.104	-1.697	0.0909
Migrant background (0="no", 1 = "yes")	0.269	0.293	-0.304	0.843	0.057	0.920	0.3582
School education > 10 years (0="no", 1 = "yes")	-0.003	0.262	-0.517	0.511	-0.001	-0.011	0.9910
Employed full time ((0="no", 1 = "yes")	-0.154	0.286	-0.715	0.407	-0.033	-0.539	0.5902
Current smoker ((0="no", 1 = "yes")	-0.131	0.285	-0.690	0.429	-0.030	-0.458	0.6475
Lifetime Cannabis use (0="no", 1 = "yes")	-0.745	0.319	-1.369	-0.120	-0.169	-2.337	0.0202
Cannabis use last 12 months (0="no", 1 = "yes")	-0.934	0.345	-1.610	-0.258	-0.195	-2.710	0.0072

Dependent Variable: Could become sluggish, no more able to cope

	Unstandardized Coefficients						
Model (n=256)	B	Std. Error			Beta	t	p
(Constant)	4.707	0.509	3.709	5.706		9.242	0.0000
Group	-0.310	0.247	-0.795	0.175	-0.079	-1.254	0.2110
Main diagnosis personality disorder (0="no", 1 = "yes")	0.272	0.453	-0.616	1.160	0.038	0.600	0.5488
Main diagnosis F4 (0="no", 1 = "yes")	0.272	0.474	-0.658	1.202	0.036	0.573	0.5672
Age in years	-0.008	0.011	-0.029	0.013	-0.051	-0.730	0.4661
Gender (0=female, 1=male)	-0.137	0.217	-0.562	0.289	-0.040	-0.629	0.5302
Migrant background (0="no", 1 = "yes")	0.387	0.284	-0.169	0.944	0.088	1.364	0.1738
School education > 10 years (0="no", 1 = "yes")	0.329	0.254	-0.168	0.826	0.084	1.296	0.1960
Employed full time ((0="no", 1 = "yes")	-0.055	0.277	-0.598	0.488	-0.013	-0.199	0.8422
Current smoker ((0="no", 1 = "yes")	0.266	0.277	-0.277	0.810	0.067	0.961	0.3374
Lifetime Cannabis use (0="no", 1 = "yes")	0.396	0.309	-0.210	1.002	0.097	1.281	0.2015

Cannabis use last 12 months (0="no", 1 = "yes")	-0.984	0.333	-1.637	-0.331	-0.223	-2.953	0.0035

Dependent Variable: Could become dependent on cannabis

	Unstandardized Coefficients		95% confidence interval				
Model (n=256)	B	Std. Error	Lower limit	Upper limit	Beta	t	p
(Constant)	5.128	0.559	4.032	6.223		9.174	0.0000
Group	0.087	0.272	-0.445	0.620	0.020	0.322	0.7481
Main diagnosis personality disorder (0="no", 1 = "yes")	0.276	0.499	-0.701	1.253	0.035	0.553	0.5805
Main diagnosis F4 (0="no", 1 = "yes")	-0.022	0.533	-1.068	1.023	-0.003	-0.042	0.9665
Age in years	-0.007	0.012	-0.030	0.016	-0.042	-0.608	0.5438
Gender (0=female, 1=male)	-0.461	0.238	-0.928	0.006	-0.122	-1.935	0.0541
Migrant background (0="no", 1 = "yes")	0.229	0.312	-0.382	0.841	0.047	0.735	0.4633
School education > 10 years (0="no", 1 = "yes")	0.265	0.279	-0.282	0.812	0.061	0.950	0.3432
Employed full time ((0="no", 1 = "yes")	0.269	0.305	-0.328	0.866	0.057	0.883	0.3781
Current smoker ((0="no", 1 = "yes")	0.512	0.304	-0.084	1.107	0.116	1.683	0.0937
Lifetime Cannabis use (0="no", 1 = "yes")	-0.725	0.339	-1.389	-0.060	-0.159	-2.138	0.0335
Cannabis use last 12 months (0="no", 1 = "yes")	-0.554	0.368	-1.275	0.168	-0.112	-1.505	0.1337

Dependent Variable: Could not work/study, concentration problems

	Unstandardized Coefficients		95% confidence interval				
Model (N=254)	B	Std. Error	Lower limit	Upper limit	Beta	t	p

(Constant)	4.643	0.518	3.628	5.658		8.965	0.0000
Group	-0.161	0.252	-0.655	0.333	-0.041	-0.639	0.5234
Main diagnosis personality disorder (0="no", 1 = "yes")	-0.217	0.472	-1.143	0.708	-0.030	-0.460	0.6459
Main diagnosis F4 (0="no", 1 = "yes")	0.293	0.482	-0.652	1.238	0.039	0.608	0.5438
Age in years	0.000	0.011	-0.021	0.022	0.001	0.018	0.9856
Gender (0=female, 1=male)	-0.208	0.221	-0.641	0.225	-0.061	-0.943	0.3466
Migrant background (0="no", 1 = "yes")	0.290	0.289	-0.277	0.858	0.066	1.003	0.3171
School education > 10 years (0="no", 1 = "yes")	0.350	0.259	-0.157	0.856	0.088	1.352	0.1775
Employed full time ((0="no", 1 = "yes")	0.075	0.283	-0.479	0.629	0.018	0.266	0.7906
Current smoker ((0="no", 1 = "yes")	0.333	0.283	-0.221	0.887	0.083	1.177	0.2405
Lifetime Cannabis use (0="no", 1 = "yes")	-0.013	0.317	-0.635	0.609	-0.003	-0.041	0.9677
Cannabis use last 12 months (0="no", 1 = "yes")	-0.696	0.339	-1.361	-0.032	-0.157	-2.055	0.0410

Dependent Variable: Non-smoker rejects joints

	Unstandardized Coefficients		95% confidence interval				
Model (n=133)	B	Std. Error	Lower limit	Upper limit	Beta	t	p
(Constant)	4.937	0.738	3.490	6.383		6.688	0.0000
Group	0.574	0.402	-0.213	1.362	0.121	1.429	0.1555
Main diagnosis personality disorder (0="no", 1 = "yes")	-0.298	0.895	-2.052	1.456	-0.028	-0.333	0.7397
Main diagnosis F4 (0="no", 1 = "yes")	-0.118	0.711	-1.512	1.276	-0.014	-0.166	0.8684
Age in years	0.014	0.016	-0.017	0.046	0.078	0.882	0.3794
Gender (0=female, 1=male)	-0.642	0.348	-1.323	0.040	-0.156	-1.845	0.0674
Migrant background (0="no", 1 = "yes")	-0.348	0.433	-1.197	0.501	-0.067	-0.802	0.4239

School education > 10 years (0="no", 1 = "yes")	-0.028	0.412	-0.835	0.778	-0.006	-0.068	0.9457
Employed full time ((0="no", 1 = "yes")	0.306	0.428	-0.533	1.144	0.061	0.715	0.4760
Lifetime Cannabis use (0="no", 1 = "yes")	-0.589	0.453	-1.476	0.299	-0.124	-1.299	0.1962
Cannabis use last 12 months (0="no", 1 = "yes")	-1.314	0.640	-2.568	-0.060	-0.202	-2.054	0.0421

Dependent Variable: Fear of being investigated by the police

Model (n=258)	Unstandardized Coefficients		95% confidence interval		Beta	t	p
	B	Std. Error	Lower limit	Upper limit			
(Constant)	5.726	0.608	4.534	6.918		9.414	0.0000
Group	-1.058	0.294	-1.635	-0.481	-0.218	-3.593	0.0004
Main diagnosis personality disorder (0="no", 1 = "yes")	0.172	0.543	-0.892	1.236	0.019	0.317	0.7519
Main diagnosis F4 (0="no", 1 = "yes")	-0.039	0.568	-1.152	1.074	-0.004	-0.068	0.9456
Age in years	-0.020	0.013	-0.045	0.005	-0.104	-1.537	0.1255
Gender (0=female, 1=male)	-0.600	0.259	-1.107	-0.093	-0.143	-2.317	0.0213
Migrant background (0="no", 1 = "yes")	0.108	0.339	-0.555	0.772	0.020	0.320	0.7489
School education > 10 years (0="no", 1 = "yes")	-0.003	0.302	-0.596	0.590	-0.001	-0.010	0.9923
Employed full time ((0="no", 1 = "yes")	0.580	0.330	-0.066	1.227	0.111	1.759	0.0798
Current smoker ((0="no", 1 = "yes")	0.343	0.330	-0.304	0.990	0.070	1.039	0.2996
Lifetime Cannabis use (0="no", 1 = "yes")	-0.678	0.368	-1.400	0.044	-0.135	-1.841	0.0668
Cannabis use last 12 months (0="no", 1 = "yes")	-0.502	0.399	-1.284	0.280	-0.092	-1.258	0.2095

Dependent Variable: Fear of problems at work/school

Model (n=258)	Unstandardized Coefficients B	Std. Error	95% confidence interval Lower limit	Upper limit	Beta	t	p
(Constant)	5.995	0.559	4.899	7.091		10.725	0.0000
Group	-0.475	0.271	-1.005	0.056	-0.105	-1.754	0.0807
Main diagnosis personality disorder (0="no", 1 = "yes")	-0.031	0.499	-1.009	0.947	-0.004	-0.062	0.9509
Main diagnosis F4 (0="no", 1 = "yes")	-0.760	0.522	-1.783	0.263	-0.088	-1.456	0.1465
Age in years	-0.024	0.012	-0.047	-0.001	-0.137	-2.034	0.0431
Gender (0=female, 1=male)	-0.497	0.238	-0.963	-0.030	-0.128	-2.088	0.0378
Migrant background (0="no", 1 = "yes")	-0.154	0.311	-0.764	0.456	-0.031	-0.496	0.6206
School education > 10 years (0="no", 1 = "yes")	0.173	0.278	-0.372	0.718	0.039	0.622	0.5348
Employed full time ((0="no", 1 = "yes")	0.855	0.303	0.260	1.449	0.176	2.819	0.0052
Current smoker ((0="no", 1 = "yes")	0.591	0.303	-0.004	1.186	0.130	1.948	0.0526
Lifetime Cannabis use (0="no", 1 = "yes")	-0.358	0.338	-1.021	0.305	-0.077	-1.057	0.2914
Cannabis use last 12 months (0="no", 1 = "yes")	-1.152	0.367	-1.871	-0.433	-0.227	-3.142	0.0019

Dependent Variable: Fear of losing driver's licence (n=159)

Model	Unstandardized Coefficients B	Std. Error	95% confidence interval Lower limit	Upper limit	Beta	t	p
(Constant)	6.122	0.728	4.696	7.549		8.411	0.0000
Group	-0.406	0.311	-1.017	0.204	-0.101	-1.305	0.1939
Main diagnosis personality disorder (0="no", 1 = "yes")	0.126	0.569	-0.990	1.243	0.017	0.222	0.8246
Main diagnosis F4 (0="no", 1 = "yes")	0.069	0.619	-1.145	1.282	0.008	0.111	0.9120

Age in years	0.005	0.013	-0.021	0.032	0.034	0.405	0.6861
Gender (0=female, 1=male)	-1.185	0.273	-1.721	-0.650	-0.337	-4.337	0.0000
Migrant background (0="no", 1 = "yes")	-0.489	0.374	-1.222	0.243	-0.103	-1.309	0.1925
School education > 10 years (0="no", 1 = "yes")	-0.083	0.313	-0.696	0.531	-0.020	-0.264	0.7925
Employed full time ((0="no", 1 = "yes")	0.355	0.306	-0.245	0.954	0.089	1.160	0.2478
Current smoker ((0="no", 1 = "yes")	0.126	0.330	-0.520	0.772	0.032	0.382	0.7032
Lifetime Cannabis use (0="no", 1 = "yes")	-0.175	0.380	-0.920	0.569	-0.042	-0.462	0.6450
Cannabis use last 12 months (0="no", 1 = "yes")	-0.559	0.412	-1.366	0.248	-0.123	-1.358	0.1764

Dependent Variable: No cannabis consumption among friends

	Unstandardized Coefficients		95% confidence interval				
Model (n=258)	B	Std. Error	Lower limit	Upper limit	Beta	t	p
(Constant)	4.116	0.591	2.957	5.275		6.961	0.0000
Group	-0.330	0.286	-0.890	0.231	-0.065	-1.153	0.2502
Main diagnosis personality disorder (0="no", 1 = "yes")	0.978	0.527	-0.054	2.010	0.105	1.857	0.0645
Main diagnosis F4 (0="no", 1 = "yes")	-0.408	0.539	-1.463	0.648	-0.043	-0.757	0.4495
Age in years	0.022	0.013	-0.002	0.047	0.111	1.765	0.0788
Gender (0=female, 1=male)	0.048	0.252	-0.446	0.541	0.011	0.189	0.8502
Migrant background (0="no", 1 = "yes")	-0.434	0.328	-1.078	0.209	-0.076	-1.322	0.1873
School education > 10 years (0="no", 1 = "yes")	0.119	0.293	-0.455	0.694	0.024	0.407	0.6841
Employed full time ((0="no", 1 = "yes")	-0.088	0.322	-0.720	0.544	-0.016	-0.273	0.7851
Current smoker ((0="no", 1 = "yes")	-0.131	0.320	-0.759	0.497	-0.025	-0.409	0.6829
Lifetime Cannabis use (0="no", 1 = "yes")	-0.349	0.357	-1.050	0.351	-0.066	-0.978	0.3289

Cannabis use last 12 months (0="no", 1 = "yes")	-2.145	0.387	-2.904	-1.386	-0.374	-5.539	0.0000

Dependent Variable: Do not want contact to drug scenes

	Unstandardized Coefficients		95% confidence interval				
Model (n=256)	B	Std. Error	Lower limit	Upper limit	Beta	t	p
(Constant)	6.092	0.475	5.161	7.023		12.823	0.0000
Group	-0.315	0.231	-0.767	0.137	-0.078	-1.366	0.1731
Main diagnosis personality disorder (0="no", 1 = "yes")	-0.113	0.423	-0.941	0.716	-0.015	-0.266	0.7904
Main diagnosis F4 (0="no", 1 = "yes")	-0.710	0.443	-1.577	0.158	-0.093	-1.603	0.1101
Age in years	0.005	0.010	-0.015	0.024	0.030	0.460	0.6456
Gender (0=female, 1=male)	-0.078	0.202	-0.475	0.318	-0.023	-0.387	0.6989
Migrant background (0="no", 1 = "yes")	-0.249	0.266	-0.770	0.272	-0.055	-0.937	0.3496
School education > 10 years (0="no", 1 = "yes")	0.219	0.236	-0.244	0.683	0.055	0.928	0.3542
Employed full time ((0="no", 1 = "yes")	0.531	0.259	0.023	1.039	0.122	2.050	0.0414
Current smoker ((0="no", 1 = "yes")	-0.223	0.257	-0.728	0.281	-0.055	-0.868	0.3863
Lifetime Cannabis use (0="no", 1 = "yes")	0.161	0.287	-0.402	0.723	0.038	0.559	0.5765
Cannabis use last 12 months (0="no", 1 = "yes")	-1.766	0.312	-2.377	-1.155	-0.389	-5.664	0.0000

Dependent Variable: I do not know where to obtain cannabis

	Unstandardized Coefficients		95% confidence interval				
Model (n=259)	B	Std. Error	Lower limit	Upper limit	Beta	t	p

(Constant)	4.875	0.580	3.738	6.013		8.403	0.0000
Group	-0.511	0.281	-1.061	0.038	-0.100	-1.823	0.0695
Main diagnosis personality disorder (0="no", 1 = "yes")	0.222	0.518	-0.793	1.237	0.024	0.428	0.6690
Main diagnosis F4 (0="no", 1 = "yes")	0.054	0.529	-0.983	1.092	0.006	0.103	0.9183
Age in years	0.015	0.012	-0.009	0.039	0.074	1.202	0.2305
Gender (0=female, 1=male)	-0.158	0.247	-0.642	0.326	-0.036	-0.639	0.5233
Migrant background (0="no", 1 = "yes")	0.041	0.321	-0.589	0.671	0.007	0.128	0.8980
School education > 10 years (0="no", 1 = "yes")	0.633	0.287	0.070	1.197	0.124	2.203	0.0285
Employed full time ((0="no", 1 = "yes")	0.766	0.315	0.150	1.383	0.138	2.437	0.0155
Current smoker ((0="no", 1 = "yes")	-0.128	0.315	-0.746	0.489	-0.025	-0.407	0.6842
Lifetime Cannabis use (0="no", 1 = "yes")	-1.245	0.351	-1.933	-0.558	-0.234	-3.549	0.0005
Cannabis use last 12 months (0="no", 1 = "yes")	-1.495	0.380	-2.241	-0.749	-0.259	-3.928	0.0001

Dependent Variable: I was never offered cannabis

	Unstandardized Coefficients		95% confidence interval				
Model (n=259)	B	Std. Error	Lower limit	Upper limit	Beta	t	p
(Constant)	3.881	0.516	2.869	4.894		7.517	0.0000
Group	-0.123	0.250	-0.612	0.367	-0.025	-0.492	0.6231
Main diagnosis personality disorder (0="no", 1 = "yes")	0.122	0.461	-0.782	1.025	0.014	0.264	0.7918
Main diagnosis F4 (0="no", 1 = "yes")	-0.485	0.471	-1.409	0.438	-0.054	-1.030	0.3038
Age in years	0.020	0.011	-0.002	0.041	0.103	1.784	0.0756
Gender (0=female, 1=male)	-0.038	0.220	-0.469	0.392	-0.009	-0.175	0.8615
Migrant background (0="no", 1 = "yes")	0.252	0.286	-0.309	0.813	0.047	0.880	0.3796

School education > 10 years (0="no", 1 = "yes")	0.001	0.256	-0.501	0.502	0.000	0.004	0.9972
Employed full time ((0="no", 1 = "yes")	0.104	0.280	-0.445	0.652	0.020	0.370	0.7115
Current smoker ((0="no", 1 = "yes")	-0.286	0.280	-0.835	0.264	-0.058	-1.019	0.3092
Lifetime Cannabis use (0="no", 1 = "yes")	-2.466	0.312	-3.078	-1.854	-0.491	-7.897	0.0000
Cannabis use last 12 months (0="no", 1 = "yes")	-0.457	0.339	-1.121	0.207	-0.084	-1.349	0.1785

Dependent Variable: It was uninteresting in my circle of friends

	Unstandardized Coefficients		95% confidence interval				
Model (n=259)	B	Std. Error	Lower limit	Upper limit	Beta	t	p
(Constant)	4.544	0.587	3.393	5.695		7.736	0.0000
Group	0.025	0.284	-0.532	0.581	0.005	0.087	0.9305
Main diagnosis personality disorder (0="no", 1 = "yes")	0.025	0.524	-1.003	1.052	0.003	0.047	0.9622
Main diagnosis F4 (0="no", 1 = "yes")	-1.194	0.536	-2.245	-0.144	-0.125	-2.229	0.0267
Age in years	0.025	0.012	0.000	0.049	0.123	1.987	0.0480
Gender (0=female, 1=male)	-0.121	0.250	-0.611	0.369	-0.027	-0.484	0.6291
Migrant background (0="no", 1 = "yes")	-0.014	0.325	-0.652	0.624	-0.002	-0.042	0.9664
School education > 10 years (0="no", 1 = "yes")	0.030	0.291	-0.540	0.600	0.006	0.103	0.9181
Employed full time ((0="no", 1 = "yes")	0.069	0.318	-0.555	0.693	0.012	0.217	0.8284
Current smoker ((0="no", 1 = "yes")	-0.921	0.319	-1.546	-0.296	-0.178	-2.889	0.0042
Lifetime Cannabis use (0="no", 1 = "yes")	-1.607	0.355	-2.303	-0.911	-0.303	-4.524	0.0000
Cannabis use last 12 months (0="no", 1 = "yes")	-0.473	0.385	-1.228	0.282	-0.082	-1.228	0.2208

Dependent Variable: One should lead one's life without the influence of drugs

Model (n=258)	Unstandardized Coefficients B	Unstandardized Coefficients Std. Error	95% confidence interval Lower limit	95% confidence interval Upper limit	Beta	t	p
(Constant)	5.523	0.452	4.637	6.410		12.218	0.0000
Group	0.234	0.219	-0.196	0.664	0.062	1.065	0.2881
Main diagnosis personality disorder (0="no", 1 = "yes")	0.465	0.404	-0.326	1.256	0.067	1.151	0.2507
Main diagnosis F4 (0="no", 1 = "yes")	-0.063	0.413	-0.872	0.746	-0.009	-0.153	0.8786
Age in years	0.015	0.010	-0.003	0.034	0.104	1.608	0.1092
Gender (0=female, 1=male)	-0.317	0.193	-0.694	0.061	-0.097	-1.644	0.1015
Migrant background (0="no", 1 = "yes")	0.068	0.251	-0.424	0.561	0.016	0.272	0.7856
School education > 10 years (0="no", 1 = "yes")	-0.345	0.224	-0.785	0.094	-0.092	-1.539	0.1250
Employed full time ((0="no", 1 = "yes")	0.217	0.247	-0.267	0.701	0.053	0.878	0.3807
Current smoker ((0="no", 1 = "yes")	-0.058	0.246	-0.540	0.423	-0.015	-0.238	0.8124
Lifetime Cannabis use (0="no", 1 = "yes")	-0.587	0.273	-1.123	-0.051	-0.150	-2.147	0.0327
Cannabis use last 12 months (0="no", 1 = "yes")	-1.055	0.297	-1.637	-0.473	-0.246	-3.553	0.0005

Dependent Variable: I do not take anything forbidden

Model (n=258)	Unstandardized Coefficients B	Unstandardized Coefficients Std. Error	95% confidence interval Lower limit	95% confidence interval Upper limit	Beta	t	p
(Constant)	6.323	0.510	5.325	7.322		12.411	0.0000
Group	0.038	0.247	-0.446	0.522	0.009	0.155	0.8772
Main diagnosis personality disorder (0="no", 1 = "yes")	0.759	0.466	-0.154	1.672	0.094	1.629	0.1047

Main diagnosis F4 (0="no", 1 = "yes")	-0.477	0.464	-1.387	0.434	-0.059	-1.026	0.3057
Age in years	-0.001	0.011	-0.022	0.020	-0.005	-0.082	0.9348
Gender (0=female, 1=male)	-0.451	0.217	-0.876	-0.027	-0.120	-2.083	0.0383
Migrant background (0="no", 1 = "yes")	0.292	0.283	-0.262	0.846	0.060	1.034	0.3021
School education > 10 years (0="no", 1 = "yes")	0.261	0.253	-0.235	0.756	0.060	1.032	0.3032
Employed full time ((0="no", 1 = "yes")	0.088	0.276	-0.454	0.630	0.019	0.318	0.7504
Current smoker ((0="no", 1 = "yes")	-0.115	0.276	-0.657	0.427	-0.026	-0.417	0.6770
Lifetime Cannabis use (0="no", 1 = "yes")	-0.210	0.309	-0.817	0.396	-0.047	-0.680	0.4973
Cannabis use last 12 months (0="no", 1 = "yes")	-1.927	0.334	-2.582	-1.272	-0.394	-5.770	0.0000

Dependent Variable: Drug education prevented me from cannabis

	Unstandardized Coefficients		95% confidence interval				
Model (n=258)	B	Std. Error	Lower limit	Upper limit	Beta	t	p
(Constant)	4.189	0.548	3.114	5.264		7.639	0.0000
Group	-0.017	0.265	-0.536	0.503	-0.004	-0.063	0.9500
Main diagnosis personality disorder (0="no", 1 = "yes")	-0.333	0.489	-1.291	0.626	-0.041	-0.680	0.4969
Main diagnosis F4 (0="no", 1 = "yes")	-0.097	0.500	-1.076	0.883	-0.012	-0.193	0.8470
Age in years	-0.011	0.012	-0.033	0.012	-0.062	-0.952	0.3420
Gender (0=female, 1=male)	-0.302	0.233	-0.758	0.154	-0.078	-1.297	0.1957
Migrant background (0="no", 1 = "yes")	0.367	0.305	-0.231	0.965	0.073	1.202	0.2304
School education > 10 years (0="no", 1 = "yes")	0.153	0.266	-0.368	0.674	0.034	0.574	0.5663
Employed full time ((0="no", 1 = "yes")	0.354	0.297	-0.228	0.935	0.073	1.192	0.2345
Lifetime Cannabis use (0="no", 1 = "yes")	-1.460	0.314	-2.076	-0.844	-0.315	-4.644	0.0000

Cannabis use last 12 months (0="no", 1 = "yes")	-0.302	0.359	-1.006	0.402	-0.060	-0.840	0.4016

Dependent Variable: Parental rejection of cannabis beheld me

	Unstandardized Coefficients		95% confidence interval				
Model (n=258)	B	Std. Error	Lower limit	Upper limit	Beta	t	p
(Constant)	5.195	0.572	4.075	6.315		9.088	0.0000
Group	-0.028	0.277	-0.571	0.514	-0.006	-0.103	0.9183
Main diagnosis personality disorder (0="no", 1 = "yes")	0.246	0.510	-0.754	1.246	0.029	0.482	0.6302
Main diagnosis F4 (0="no", 1 = "yes")	-1.023	0.533	-2.068	0.022	-0.116	-1.918	0.0563
Age in years	-0.022	0.012	-0.046	0.001	-0.124	-1.855	0.0647
Gender (0=female, 1=male)	-0.126	0.243	-0.603	0.351	-0.031	-0.516	0.6063
Migrant background (0="no", 1 = "yes")	0.108	0.317	-0.513	0.730	0.021	0.342	0.7325
School education > 10 years (0="no", 1 = "yes")	-0.428	0.284	-0.984	0.127	-0.093	-1.511	0.1321
Employed full time ((0="no", 1 = "yes")	0.652	0.310	0.044	1.260	0.130	2.101	0.0366
Current smoker ((0="no", 1 = "yes")	-0.720	0.311	-1.330	-0.111	-0.154	-2.317	0.0213
Lifetime Cannabis use (0="no", 1 = "yes")	-1.005	0.346	-1.683	-0.326	-0.210	-2.903	0.0040
Cannabis use last 12 months (0="no", 1 = "yes")	-0.324	0.376	-1.061	0.412	-0.062	-0.863	0.3892

Dependent Variable: I observed others becoming sluggish

	Unstandardized Coefficients		95% confidence interval				
Model (n=256)	B	Std. Error	Lower limit	Upper limit	Beta	t	p

(Constant)	4.269	0.627	3.040	5.497		6.812	0.0000
Group	0.107	0.305	-0.490	0.705	0.022	0.352	0.7253
Main diagnosis personality disorder (0="no", 1 = "yes")	-0.610	0.559	-1.707	0.486	-0.068	-1.092	0.2761
Main diagnosis F4 (0="no", 1 = "yes")	-0.509	0.586	-1.656	0.639	-0.054	-0.869	0.3858
Age in years	-0.023	0.013	-0.049	0.003	-0.119	-1.738	0.0835
Gender (0=female, 1=male)	-0.421	0.267	-0.945	0.103	-0.098	-1.575	0.1165
Migrant background (0="no", 1 = "yes")	-0.499	0.350	-1.185	0.187	-0.090	-1.426	0.1551
School education > 10 years (0="no", 1 = "yes")	0.069	0.314	-0.546	0.684	0.014	0.218	0.8273
Employed full time ((0="no", 1 = "yes")	0.074	0.341	-0.595	0.743	0.014	0.218	0.8275
Current smoker ((0="no", 1 = "yes")	0.334	0.342	-0.337	1.005	0.067	0.976	0.3300
Lifetime Cannabis use (0="no", 1 = "yes")	1.080	0.380	0.334	1.825	0.209	2.839	0.0049
Cannabis use last 12 months (0="no", 1 = "yes")	0.177	0.413	-0.633	0.987	0.032	0.429	0.6685

Dependent Variable: Observed how others got into trouble with the police

	Unstandardized Coefficients		95% confidence interval				
Model (n=257)	B	Std. Error	Lower limit	Upper limit	Beta	t	p
(Constant)	3.751	0.651	2.475	5.027		5.762	0.0000
Group	-0.454	0.316	-1.073	0.165	-0.086	-1.437	0.1520
Main diagnosis personality disorder (0="no", 1 = "yes")	-0.176	0.581	-1.315	0.963	-0.018	-0.303	0.7620
Main diagnosis F4 (0="no", 1 = "yes")	0.107	0.608	-1.085	1.299	0.011	0.176	0.8602
Age in years	-0.024	0.014	-0.051	0.003	-0.116	-1.724	0.0859
Gender (0=female, 1=male)	-0.107	0.277	-0.650	0.437	-0.024	-0.385	0.7008
Migrant background (0="no", 1 = "yes")	0.741	0.363	0.030	1.452	0.127	2.043	0.0422

School education > 10 years (0="no", 1 = "yes")	-0.402	0.325	-1.039	0.235	-0.077	-1.236	0.2177
Employed full time ((0="no", 1 = "yes")	-0.266	0.355	-0.961	0.429	-0.047	-0.750	0.4538
Current smoker ((0="no", 1 = "yes")	0.497	0.354	-0.196	1.190	0.094	1.405	0.1614
Lifetime Cannabis use (0="no", 1 = "yes")	1.132	0.395	0.359	1.906	0.208	2.868	0.0045
Cannabis use last 12 months (0="no", 1 = "yes")	0.029	0.427	-0.808	0.867	0.005	0.069	0.9452

Dependent Variable: Observed how others developed a psychosis

	Unstandardized Coefficients		95% confidence interval				
Model (n=257)	B	Std. Error	Lower limit	Upper limit	Beta	t	p
(Constant)	2.274	0.625	1.049	3.499		3.638	0.0003
Group	0.100	0.303	-0.494	0.695	0.021	0.331	0.7410
Main diagnosis personality disorder (0="no", 1 = "yes")	-0.491	0.558	-1.584	0.602	-0.056	-0.881	0.3794
Main diagnosis F4 (0="no", 1 = "yes")	-0.023	0.584	-1.167	1.122	-0.002	-0.039	0.9693
Age in years	0.007	0.013	-0.019	0.033	0.036	0.512	0.6088
Gender (0=female, 1=male)	-0.273	0.266	-0.795	0.249	-0.066	-1.026	0.3060
Migrant background (0="no", 1 = "yes")	0.570	0.348	-0.112	1.252	0.107	1.637	0.1029
School education > 10 years (0="no", 1 = "yes")	0.085	0.312	-0.527	0.696	0.018	0.272	0.7862
Employed full time ((0="no", 1 = "yes")	-0.394	0.340	-1.061	0.273	-0.076	-1.157	0.2486
Current smoker ((0="no", 1 = "yes")	-0.128	0.340	-0.794	0.537	-0.027	-0.378	0.7061
Lifetime Cannabis use (0="no", 1 = "yes")	0.470	0.379	-0.272	1.213	0.095	1.242	0.2155
Cannabis use last 12 months (0="no", 1 = "yes")	0.214	0.410	-0.590	1.018	0.040	0.521	0.6029
