# Supplementary material for: Attitudes of Patients with Non-Psychotic Mental Disorders Towards Cannabis After Its Legalization—Comparison with Patients Before Legalization
Source: Brain Sci. 2026 Jul 11;16(7):730. doi: 10.3390/brainsci16070730 (PMC13406209; doi:10.3390/brainsci16070730)
Supplement: Supplementary file 1 [file brainsci-16-00730-s001.zip › S4 Comparisons of attitudes between recent cannabis users and nonusers.pdf]

Supplement 4: Comparison between last year's cannabis users and nonusers with regard to their attitudes toward cannabis.

Item	Used Cannabis during the last 12 month	N	Mean	SD	t	d.f.	p[1]	Mean difference	s.e. of mean difference	95% c.i. lower bound	95% c.i. upper bound
**Health and performance concerns**											
I could become mentally ill, or existing mental health issues could get worse.	Did use	69	3.7	2.17	-3.24	111.4	.002	-.97	.308	-1.56	-.38
	Did not use	188	4.7	1.97							
I might start using hard drugs like heroin or cocaine.	Did use	69	1.8	1.52	-2.75	164.8	.018	-.65	.237	-1.12	-.18
	Did not use	188	2.4	2.07							
I could get physically ill from consuming cannabis.	Did use	69	2.4	1.71	-5.42	150.6	< .001	-1.40	.258	-1.91	-.89
	Did not use	187	3.8	2.13							
I could become sluggish and no longer manage my life properly.	Did use	69	4.2	2.06	-2.20	112.8	.030	-.63	.284	-1.19	-.06
	Did not use	186	4.8	1.89							
I could become dependent on cannabis eventually.	Did use	68	4.0	2.7	-2.73	117.1	.007	-.83	.306	-1.44	-.23
	Did not use	187	4.9	2.13							
I could not concentrate properly at school/university/work anymore and I quickly forget what I've learned.	Did use	69	4.3	2.04	-2.36	115.4	.020	-.67	.283	-1.23	-.11
	Did not use	184	4.9	1.91							
As a non-smoker, I avoid smoking cannabis cigarettes (joints).	Did use	21	3.1	2.31	-4.12	27.3	< .001	-2.25	.546	-3.37	-1.13
	Did not use	111	5.3	2.20							
**Legal and social problems**											
I would be afraid that the police might catch me consuming cannabis and then launch an investigation against me.	Did use	69	3.7	2.58	-1.77	111.5	.080	-.63	.354	-1.33	.075
	Did not use	188	4.3	2.34							
I'd be worried about getting in trouble at school / university / work if I got caught with cannabis.	Did use	69	3.9	2.32	-3.56	112.2	< .001	-1.14	.320	-1.77	-.50
	Did not use	188	5.0	2.12							

I would be afraid of losing my driver's license if I got caught with cannabis in traffic.	Did use	39	4.9	2.35	-2.03	52.9	.048	-.83	.410	-1.65	-.01
	Did not use	121	5.7	1.79							
**Social Distance to Cannabis and Cannabis Users**											
As far as I know, no one in my circle of friends uses cannabis.	Did use	69	2.0	1.75	-9.55	166.5	< .001	-2.63	.275	-3.17	-2.08
	Did not use	188	4.6	2.42							
I do not want to have any contact with illegal drug scenes or dealers to obtain cannabis.	Did use	68	4.4	2.44	-6.05	86.3	< .001	-1.91	.316	-2.54	-1.28
	Did not use	187	6.3	1.51							
I wouldn't even know how to obtain cannabis regularly.	Did use	69	2.7	2.33	-7.32	120.6	< .001	-2.39	.327	-3.04	-1.75
	Did not use	189	5.1	2.32							
Cannabis was never offered to me.	Did use	69	1.4	1.11	-8.41	248.9	< .001	-1.93	.229	-2.38	-1.48
	Did not use	189	3.3	2.56							
Cannabis wasn't interesting to me as a teenager and young adult because nobody in my circle of friends used it.	Did use	69	2.48	2.13	-5.79	142.5	< .001	-1.82	.315	-2.45	-1.20
	Did not use	189	4.30	2.53							
**Rejection of Drugs**											
I think people should live their lives without the influence of drugs.	Did use	68	4.3	1.95	-5.86	105.3	< .001	-1.56	.267	-2.09	-1.03
	Did not use	189	5.7	1.69							
I do not take drugs that are prohibited in our country.	Did use	69	4.3	2.59	-6.33	88.8	< .001	-2.12	.334	-2.78	-1.45
	Did not use	188	6.3	1.64							
**Educational Influences**											
I was kept from using cannabis by drug education at school and in the media.	Did use	69	2.2	1.947	-3.39	140.6	< .001	-.97	.287	-1.54	-.41
	Did not use	188	3.2	2.279							
I was kept from using cannabis by my parents' clear disapproval.	Did use	69	2.7	2.09	-2.72	135.3	.007	-.83	.305	-1.43	-.23
	Did not use	188	3.5	2.35							
**Observation of negative consequences**											
	Did use	68	4.7	2.24	2.41	133.2	0.017	.79	.328	.14	1.44

I noticed how someone else (friend, acquaintance, classmate, colleague, family member) became sluggish from using cannabis and was no longer able to handle their tasks.	Did not use	187	3.9	2.53							
I witnessed someone else (friend, acquaintance, classmate, colleague, family member) get into trouble with the police because of cannabis use.	Did use	69	2.5	2.13	2.97	119.3	.004	1.09	.367	.36	1.81
	Did not use	189	4.3	2.53							
I noticed someone else (friend, acquaintance, classmate, coworker, family member) became psychotic from cannabis use (e.g., with hallucinations, delusions).	Did use	69	4.3	2.62	1.05	117.1	.29	.36	.343	-.32	1.041
	Did not use	187	3.3	2.56							

[1]Welch t-test
